# Supplementary material for: Trends of prescribing antimicrobial drugs for urinary tract infections in primary care in the Netherlands: a population-based cohort study
Source: BMJ Open. 2019 May 19;9(5):e027221. doi: 10.1136/bmjopen-2018-027221 (PMC6530323; doi:10.1136/bmjopen-2018-027221)
Supplement: Supplementary file 1 [file bmjopen-2018-027221supp001.pdf]

Supplementary table 1: ATC codes of antimicrobial drugs prescribed by GPs to treat urinary tract infections

ATC code	Drug class
J01AA*	Tetracyclines
J01CA	Penicillins with extended spectrum
J01CE*	Beta-lactamase sensitive penicillins
J01CF*	Beta-lactamase resistant penicillins
J01CR	Combinations of penicillins, incl. beta-lactamase inhibitors
J01DB*	First-generations cephalosporins
J01DC*	Second-generation cephalosporins
J01DD*	Third-generation cephalosporins
J01EA	Trimethoprim and derivatives
J01EB*	Short-acting sulfonamides
J01EE	Combinations of sulfonamides, and trimethoprim, incl. derivatives
J01FA*	Macrolides
J01FF*	Lincosamides
J01GB*	Other aminoglycosides
J01MA	Fluoroquinolones
J01MB*	Other quinolones
J01XC*	Steroid antibacterials
J01XE	Nitrofuran derivatives
J01XX	Other antibacterials

All antimicrobial drug classes that were prescribed for UTIs in the study population. The number of prescriptions of drugs with * was very low and are not shown in the figures.

Supplementary table 2: ICPC codes

ICPC code	Indication
A03	Fever
A78	Lyme disease
A99	Other generalized/not specified diseases
B70	Acute lympadenitis
D70	Infectious diarrhea
D73	Supposititious gastroenteritis
F70	Infectious conjunctivitis
F72	Blepharitis
F73	Other infection/infection eye/adnexa
H04	Excretion from ear
H70	Otitis externa
H71	Acute otitis media
H72	Otits media with effusion
H74	Chronic otitis media/mastoiditis/other infection
R02	Dyspnoea attributed to airways
R05	Cough
R09	Symptoms/complaints sinuses
R21	Sore throat
R22	Symptoms/complaints tonsils
R70	Tuberculosis (airways)
R71	Pertussis
R72	Scarlet fever
R74	Cold/pharyngitis
R75	Acute/chronic rhinosinusitis
R76	Acute tonsillitis/peritonsillar abscess
R77	Acute laryngitis/tracheitis
R78	Acute bronchitis
R81	Pneumoniae
R83	Other respiratory infections
R90	Hypertrophy/chronic infection tonsils
S06	Local redness/erythema skin
S09	Local infection finger/toe/paronychia
S10	Furuncle/karbunkel/local cellulitis
S11	Folliculitis

S12	Tick bite
S13	Bite human/animal
S18	Cut
S76	Other skin infection
S84	Impetigo
S87	Constitutional eczema
S88	Other eczema
S96	Acne
U01	Painful micturition*
U02	Frequent micturition*
U70	Acute pyelonephritis*
U71	Cystitis*
U72	Urethritis
U88	Glomerulonephritis
X84	Vaginitis
X99	Other disease of female reproduction organs
W94	Mastitis
Y74	Orchitis/Epididymitis
Y75	Balanitis

All indications that were used in the study as possible indications for which antimicrobial drugs were prescribed. Antimicrobial drugs prescribed for the indications with * were assigned as antimicrobial drugs prescribed for UTIs.

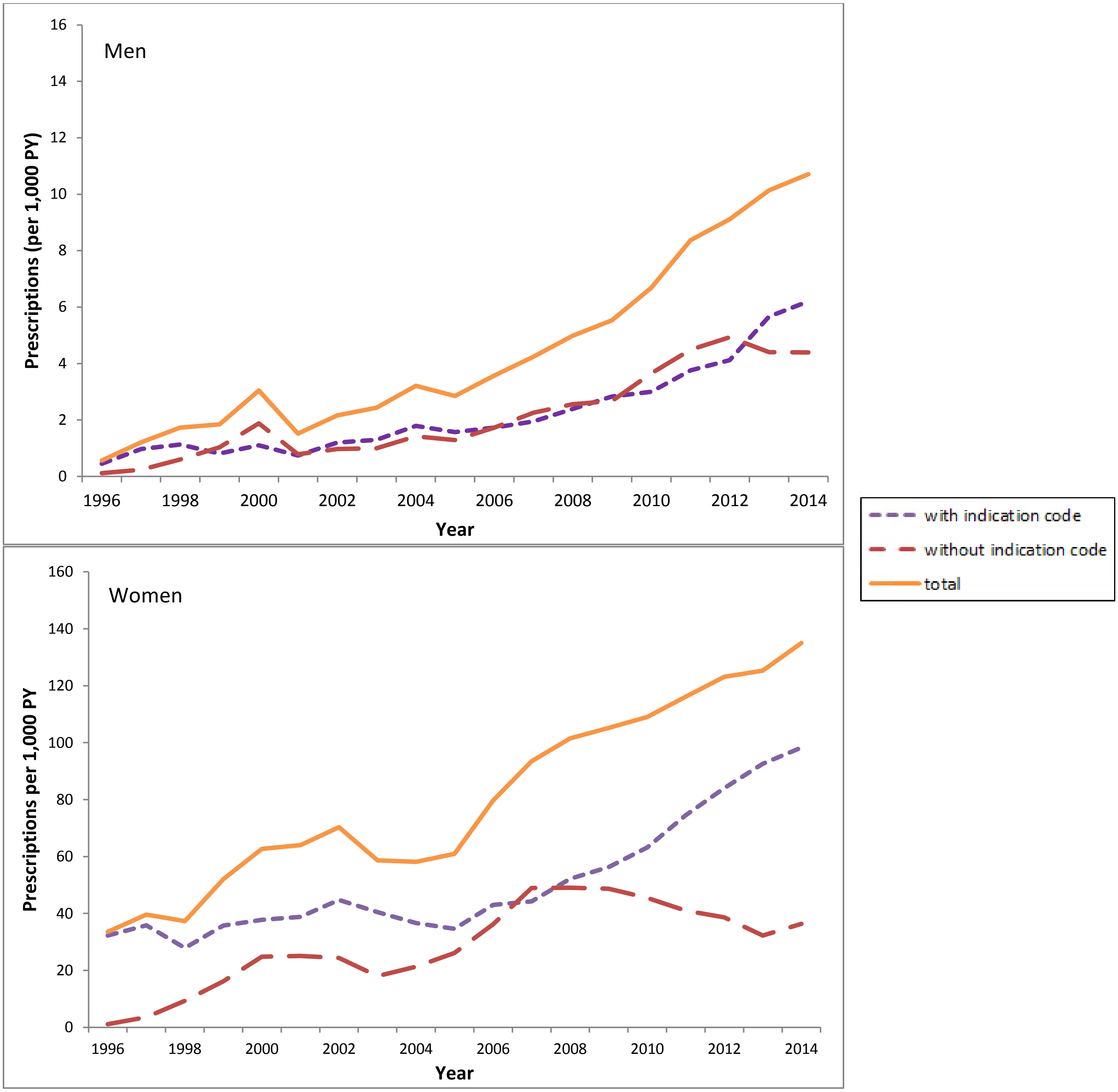

Supplementary figure 1: The number of prescriptions of nitrofurantoin with and without an indication code for urinary tract infections for men and women. Note that the scale of the y-axis differs between men and women.

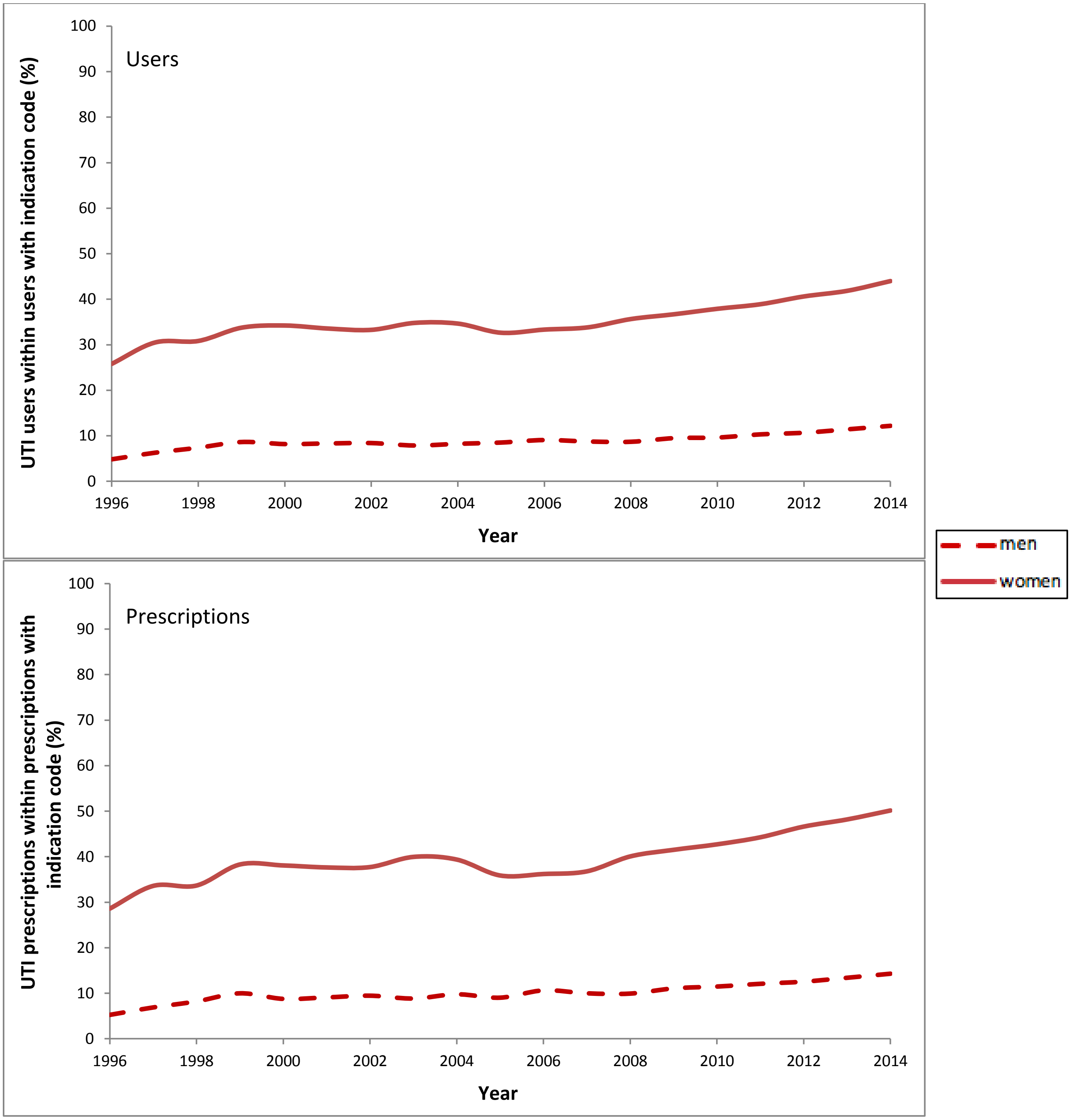

Supplementary figure 2: The percentage of users/prescriptions with an indication code for UTIs within all users/prescriptions with an indication code (thus including indication codes for UTIs, airway infections, ear infections, skin infections etc.).

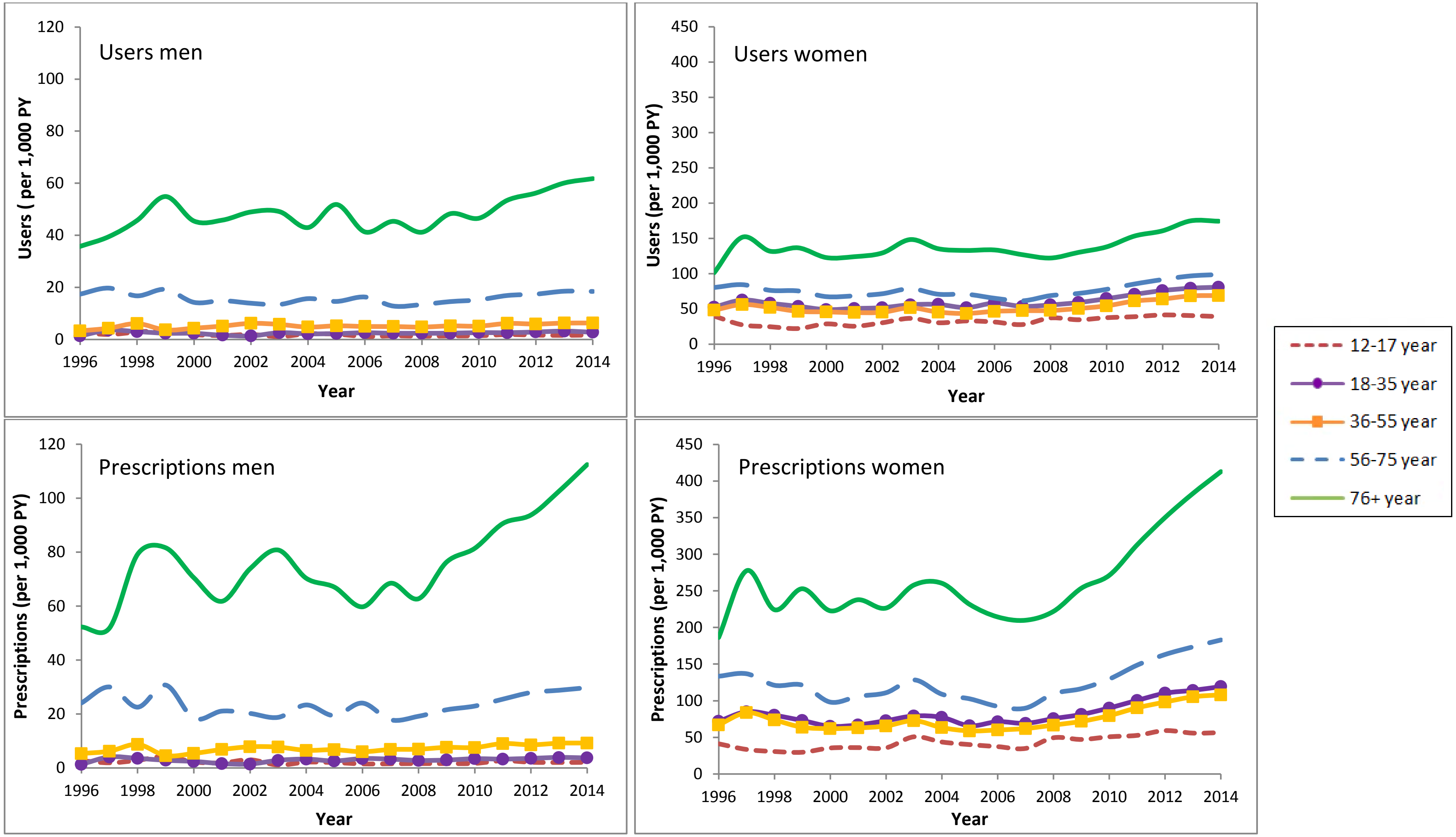

Supplementary figure 3: The number of users and prescriptions of antimicrobial drugs for urinary tract infections for different age groups per year. Note that the scale of the y-axis differs between men and women.
