# Supplementary material for: Trends of prescribing antimicrobial drugs for urinary tract infections in primary care in the Netherlands: a population-based cohort study
Source: BMJ Open. 2019 May 19;9(5):e027221. doi: 10.1136/bmjopen-2018-027221 (PMC6530323; doi:10.1136/bmjopen-2018-027221)
Supplement: Author's manuscript [file bmjopen-2018-027221.draft_revisions.pdf]

BMJ Open is committed to open peer review. As part of this commitment we make the peer review history of every article we publish publicly available.

When an article is published we post the peer reviewers' comments and the authors' responses online. We also post the versions of the paper that were used during peer review. These are the versions that the peer review comments apply to.

The versions of the paper that follow are the versions that were submitted during the peer review process. They are not the versions of record or the final published versions. They should not be cited or distributed as the published version of this manuscript.

BMJ Open is an open access journal and the full, final, typeset and author-corrected version of record of the manuscript is available on our site with no access controls, subscription charges or pay-per-view fees (http://bmjopen.bmj.com).

If you have any questions on BMJ Open's open peer review process please email editorial.bmjopen@bmj.com

BMJ Open

Trends of prescribing antimicrobial drugs for urinary tract infections in primary care in the Netherlands

Journal:	*BMJ Open*
Manuscript ID	bmjopen-2018-027221
Article Type:	Research
Date Submitted by the Author:	12-Oct-2018
Complete List of Authors:	Mulder, Marlies; Erasmus MC, Epidemiology<br>Baan, Esme; Erasmus MC,<br>Verbon, Annelies ; Erasmus MC, Microbiology and Infectious diseases<br>Stricker, Bruno; Ersamus MC, Drug Safety Unit<br>Verhamme, Katia; Erasmus Medical Center
Keywords:	Urinary tract infections < UROLOGY, EPIDEMIOLOGY, PRIMARY CARE

Trends of prescribing antimicrobial drugs for urinary tract infections in primary care in the Netherlands

Running title: Prescribing of antimicrobial drugs for UTIs by GPs in the Netherlands

M. Mulder[1,2], E.J. Baan[3], A. Verbon[5], B.H.C. Stricker[1,2,5], K.M.C. Verhamme[3,6]

1. Department of Epidemiology, Erasmus Medical Centre, PO Box 2040, 3000 CA Rotterdam, The Netherlands

2. Health and Youth Care Inspectorate, Postbus 2518, 6401 DA Heerlen, The Netherlands

3. Department of Medical Informatics, Erasmus Medical Centre, PO Box 2040, 3000 CA Rotterdam, The Netherlands

4. Department of Medical Microbiology and Infectious Diseases, Erasmus Medical Centre, PO Box 2040, 3000 CA Rotterdam, The Netherlands

5. Department of Internal Medicine, Erasmus Medical Centre, PO Box 2040, 3000 CA Rotterdam, The Netherlands

6. Department of Bioanalysis, Faculty of Pharmaceutical Sciences, University of Ghent, Belgium

Keywords: antibiotic stewardship, antimicrobial drug prescribing, urinary tract infections, primary care.

Address for Correspondence:
Professor Bruno H.Ch. Stricker
Department of Epidemiology
Erasmus MC - University Medical Center Rotterdam
P.O. Box 2040
3000 CA Rotterdam
The Netherlands
b.stricker@erasmusmc.nl

For peer review only - http://bmjopen.bmj.com/site/about/guidelines.xhtml

Abstract

Objective: Urinary tract infections (UTIs) are an important reason to consult a General Practitioner (GP) in the Netherlands. In this study, we describe antimicrobial drug prescribing patterns for UTIs and investigate adherence of prescribers to Dutch primary care guidelines.

Methods: We conducted a population-based cohort study in IPCI, a Dutch GP database encompassing approximately 2.5 million patients. All antimicrobial drug prescriptions to patients ≥12 years in 1996-2014 were extracted. The number of prescriptions and choice of drug type were investigated over calendar time and in different age categories. The choice of antimicrobial drugs classes for UTIs and the duration of nitrofurantoin use in women were compared to the Dutch primary care guidelines.

Results: The source population comprised 1,755,085 patients who received 2,019,335 antimicrobial drug prescriptions; 401,655 (35.1%) prescriptions were for UTIs (45.2% in women; 12.6% in men). The proportion of prescriptions for UTI within all prescriptions with an indication code increased from 5.2% in 1996 to 14% in 2014 in men and from 28% in 1996 to 50% in 2014 for women. In men, UTIs were most frequently treated with fluoroquinolones during the entire study period, whereas use of fluoroquinolones was only advised as a first-choice in the latest guideline of 2013. In women, UTIs were increasingly ($p<0.05$) treated with nitrofuran derivatives with a significant effect of the guideline of 2005 ($p<0.05$). Adherence to the advised duration of nitrofurantoin prescriptions in women has increased since the guideline of 2005.

Conclusions: The antimicrobial drug prescribing for UTIs seem to have increased over time. Adherence to the UTI guidelines increased with regard to choice and duration of antimicrobial drugs. We showed that databases like IPCI in which prescription and indication are monitored, can be valuable antibiotic stewardship tools.

For peer review only

For peer review only - http://bmjopen.bmj.com/site/about/guidelines.xhtml

Strengths and limitations of this study

- This study was conducted in a large database, including a substantial part (~15%) of the Dutch population implying that the external validity of our study is high.
- In the Netherlands, every person is assigned to a specific GP which limits selection bias.
- Improvement of coding over calendar time might bias conclusions on total prescribing of antimicrobial drugs. Therefore, we also studied the prescription data for other infections to be able to study prescriptions of antimicrobial drugs for UTIs in comparison with prescriptions of antimicrobial drugs for other infections
- We analyzed the adherence of GPs to the Dutch guidelines on treatment of urinary tract infections and show that database studies can be used as a tool for good antimicrobial stewardship.

For peer review only

For peer review only - http://bmjopen.bmj.com/site/about/guidelines.xhtml

Introduction

Urinary tract infections (UTIs) are among the most common infections in humans. UTIs cause a substantial burden of disease with major economic consequences.[1 2] In women, UTIs are the most common reason to consult a general practitioner (GP) in the Netherlands with 232 contacts per 1,000 women in 2014. In men, this number was substantially lower: 37 contacts per 1,000 men.[3]

In most cases UTIs are treated with antimicrobial drugs. The choice of antimicrobial drug depends on the antimicrobial sensitivity of pathogens in urinary cultures (if taken), severity of the symptoms and potential co-morbidities of the individual. Based on the resistance patterns of *E. coli* in the Netherlands, Dutch guidelines on the treatment of UTIs have changed several times in the last few decades. The latest Dutch guideline for the treatment of UTIs in primary care was released in 2013 and recommends nitrofurantoin as first-choice in all patients with cystitis, including men. In case of signs of tissue invasion, ciprofloxacin is recommended as first-choice.[4] Additionally, the recommended duration of treatment has changed. The recommended duration of treatment with nitrofurantoin for cystitis in women was 3 days in the guidelines of 1989 and 1999, whereas it has been extended to 5 days in the guidelines of 2005 and 2013 (**Table 1**).[4-7]

Previous research shows that the use of antimicrobial drugs in the Netherlands in general has increased since 2005 until approximately 2012, most prominently in elderly patients, but has decreased again since then.[8 9] Since UTIs are a major reason to consult a GP, use of antimicrobial drugs for UTIs contribute significantly to total antimicrobial consumption. Therefore, antibiotic stewardship focusing on (inappropriate) antimicrobial drug prescribing for UTIs is an important target in the fight against antimicrobial resistance (AMR). This study aims to use a large GP database to investigate the adherence of GPs to the Dutch UTI guidelines, by studying choice of antimicrobial drug and duration of nitrofurantoin use over time.

For peer review only - http://bmjopen.bmj.com/site/about/guidelines.xhtml

For peer review only

Table 1: Overview of the treatment of UTIs according to the Dutch guidelines

	1989	1999	2005	2013
**Women**				
Cystitis	Trimethoprim (J01EA) *or* Sulfamethizol (J01EB) *or* Nitrofurantoin (J01XE) ***3 days***	Nitrofurantoin (J01XE) ***3 days*** *or* Trimethoprim (J01EA)	*1st choice*: Nitrofurantoin (J01XE) ***5 days***<br>*2nd choice*: Trimethoprim (J01EA)<br>*3rd choice*: Fosfomycin (J01XX)	*1st choice*: Nitrofurantoin (J01XE) ***5 days***<br>*2nd choice*: fosfomycin (J01XX)<br>*3rd choice*: Trimethoprim (J01EA)
UTI with tissue invasion	Amoxicillin (J01CA)	*1st choice*: Amoxicillin-clavulanic acid (J01CR)<br>*2nd choice*: Sulfamethoxazole-trimethoprim (J01EE)	*1st choice*: Amoxicillin-clavulanic acid (J01CR)<br>*2nd choice*: Sulfamethoxazole-trimethoprim (J01EE) *or* Fluoroquinolone (J01MA)	*1st choice*: Ciprofloxacin (J01MA)<br>*2nd choice*: Amoxicillin-clavulanic acid (J01CR)<br>*3rd choice*: Sulfamethoxazole-trimethoprim (J01EE)
**Men**				
Cystitis	UTI in men should always be considered to be prostatitis	Nitrofurantoin (J01XE) *or* Trimethoprim (J01EA)	*1st choice*: Nitrofurantoin (J01XE)<br>*2nd choice*: Trimethoprim (J01EA)	*1st choice:* Nitrofurantoin (J01XE)<br>*2nd choice*: Trimethoprim (J01EA)
UTI with tissue invasion	Trimethoprim (J01EA) *or* Amoxicillin (J01CA)	*1st choice*: Amoxicillin-clavulanic acid (J01CR)<br>*2nd choice*: sulfamethoxazole-trimethoprim (J01EE)	*1st choice*: Amoxicillin-clavulanic acid (J01CR)<br>*2nd choice*: Sulfamethoxazole-trimethoprim (J01EE) *or* Fluorochinolone (J01MA)	*1st choice*: Ciprofloxacin (J01MA)<br>*2nd choice*: Amoxicillin-clavulanic acid (J01CR)<br>*3rd choice*: Sulfamethoxazole-trimethoprim (J01EE)

The tabel shows the treatment of UTIs according to the Dutch guidelines of 1989, 1999, 2005 and 2013.[4-7] The recommended duration of treatment with nitrofurantoin is also described. Please note that information from guidelines on the treatment of pregnant women, and also the treatment of risk groups such as diabetes patients or abnormalities of the urinary tract differs is not described. Furthermore, the treatment duration of men is in all case longer than in women.

For peer review only - http://bmjopen.bmj.com/site/about/guidelines.xhtml

Materials and methods

Study population

This study was conducted using data from the Integrated Primary Care Information database (IPCI), which is a longitudinal observational dynamic database containing the records from more than 450 general practices throughout the Netherlands.[10] Briefly, IPCI contains the complete electronic medical records of ~2,500,000 patients, containing amongst others data on diagnoses (coded and free text) and prescriptions coded according to the ATC (anatomical therapeutical chemical) classification of the WHO.[11] The system complies with European Union guidelines on the use of data for medical research and has been proven valid for pharmaco-epidemiological studies. More detailed information on IPCI has been described elsewhere.[10] The scientific and ethical advisory board of IPCI approved this study (nr. 11/2015).

The study cohort comprised patients aged 12 years and older with at least one year of valid database history in the IPCI database. The study period was from the 1st of January 1996 until the 1st of January 2015. Follow-up started from the following, whichever occurred last: start of study period, age of 12 years, and reaching a minimum of 12 months of database history. Follow-up ended when a patient left the database, died or at the end of the study period, whichever occurred first. Gender and age at time of prescription(s) were assessed for all patients.

Prescriptions

From the database, we selected all prescriptions for antimicrobial drugs prescribed during the study period, using an automatic search on the ATC code "J01", which is the ATC code for antimicrobial drugs. All prescriptions of antimicrobial drugs were further categorized by ATC drug class e.g. J01AA (tetracyclines). (**Table S1**) We analyzed all prescriptions of ATC class J01CA (penicillins with extended

For peer review only - http://bmjopen.bmj.com/site/about/guidelines.xhtml

112 spectrum), J01CR (combinations of penicillins, incl. beta-lactamase inhibitors), J01EA (trimethoprim and
113 derivatives), J01EE (combinations of sulfonamides and trimethoprim, incl. derivatives), J01MA
114 (fluoroquinolones), J01XE (nitrofuran derivatives) and J01XX (other antimicrobials (mainly fosfomycin)).
115 In addition, the duration of all prescriptions of nitrofurantoin (J01XE01) (which in the Netherlands is only
116 prescribed for UTIs) was assessed.

117 *Indication of use of antimicrobial drugs*

118 Indication of use was assessed through an automatic search on disease specific codes. Antimicrobial
119 drug prescriptions were linked to the indication of use through a unique patient identifier linking a
120 prescription to a diagnosis using the International Classification of Primary Care (ICPC) codes. These ICPC
121 codes were categorized in the following indications: UTIs, skin infections, respiratory infections, ear
122 infections or other infections. All prescriptions without an ICPC code were assigned to the group: 'no
123 code for indication of use'. Urethritis was included in the group of "other" infections and not in the UTI
124 group because of the distinct pathophysiology.

125 *Analyses*

126 The total number of prescriptions for UTIs, 'other infections' (including respiratory infections, skin
127 infections, ear infections etc.) and prescriptions without an indication code were calculated for the
128 complete study time and per year. Additionally, the proportion of UTI prescriptions per calendar year
129 was calculated with as denominator all antimicrobial drug prescriptions with an indication code. Also,
130 all nitrofurantoin prescriptions were analyzed per year.

131 Because of the dynamic nature of the study cohort, the annual prevalence of antimicrobial drug
132 prescriptions was calculated by dividing the total number of antimicrobial drug prescriptions by the total
133 number of person-years (PY). Furthermore, the annual number of users per calendar year was

For peer review only - http://bmjopen.bmj.com/site/about/guidelines.xhtml

calculated by dividing all users by the total number of PY in that specific calendar year. With regard to the calculation of users, if an individual received prescriptions of more than one antimicrobial drug classes in one calendar year, the individual contributed data to the different classes. However, if an individual received 2 or more prescriptions of the same drug in one calendar year, the individual contributed only once as a user of this specific drug class. The prevalence of prescriptions and the prevalence of users were studied by age (12-17, 18-25, 26-35, 36-45, 46-55, 56-65, 66-75, 76-85 and ≥86 years age categories), gender and calendar year.

Finally, all nitrofurantoin prescriptions prescribed to women during the study period were selected and duration of use was categorized in 3, 5, 7 days or in "other" (when the number of days of use was unknown or different to 3, 5, or 7 days). The proportion of each category with as denominator the total number of prescriptions of nitrofurantoin in women was calculated by calendar year.

ARIMA modeling using SPSS 24 was performed according to a previously proposed method.[12] Calendar time was first univariately added to the analysis in order to study the trends of the different antimicrobial drug groups over time. Furthermore, a model including calendar time, intervention and interaction was performed to investigate whether there was a significant difference in slope (interaction) as an effect of the implementation of the guideline of 2005.

For peer review only - http://bmjopen.bmj.com/site/about/guidelines.xhtml

Results

The study population comprised 1,755,085 patients of 12 years and older with a mean follow-up time of 3.31 year. 671,251 (38.2%) patients were prescribed at least one antimicrobial drug during the study period; 271,772 (40.5%) men and 399,479 (59.5%) women. In total, they received 2,019,335 antimicrobial drug prescriptions (mean of 3.0 prescriptions per person) during the study period. Of these prescriptions, 1,144,810 (56.7%) could be linked to an indication, namely 528,464 (46.2%) antimicrobial drug prescriptions for airway infections; 401,655 (35.1%) for UTIs; 157,900 (13.8%) for skin infections; 29,984 (2.6%) for ear infections and 26,807 (2.3%) for "other" infections.

The subsequent sections describe the prescribing of antimicrobial drugs per calendar year and age category for both men and women separately. To study the adherence of GPs to the national guidelines as a tool in antibiotic stewardship, the results are compared to the recommendations according to the national GP guidelines **(Table 1)**.[4-7]

Antimicrobial drug prescriptions for UTIs by calendar year

For all years combined and only considering those prescriptions with indication of use, prescriptions for UTIs were 12.6% of the total prescriptions in men and 45.2% in women. The total number of prescriptions per year for UTIs increased from 9 (men) and 88 (women) prescriptions per 1,000 PY in 1996 to respectively 19 and 153 prescriptions per 1,000 PY in 2014. However, especially at the end of the study period, prescriptions without indication code decreased **(Figure 1)**. To control for improvement of coding over calendar time, we studied the total number of nitrofurantoin prescriptions, which in the Netherlands is only prescribed for the indication UTI. In women more clearly than in men, we see a flattening or even decrease of the number of non-coded nitrofurantoin prescriptions, whereas the number of coded and total nitrofurantoin prescriptions increased **(Figure S1)**. Moreover, we calculated the proportion of AB prescriptions for UTI with as denominator all prescriptions with an

For peer review only - http://bmjopen.bmj.com/site/about/guidelines.xhtml
For peer review only

indication of use. In this analysis, the proportion of AB for UTIs increased from 5.2% in 1996 to 14% in 2014 for men and from 28% in 1996 to 50% in 2014 for women **(Figure S2)**.

Fluoroquinolones (J01MA) are the most commonly prescribed antimicrobial drugs in men with UTIs with no clear increase or decrease over time. Other frequently prescribed antimicrobial drugs were combinations of sulfonamides and trimethoprim (J01EE) and combinations of penicillins, incl. beta-lactamase inhibitors (J01CR), the last group increased significantly over time until 2013. Also, in men, the number of nitrofuran derivatives prescriptions significantly increased from 0.4 prescriptions per 1,000 PY in 1996 to 6.2 prescriptions per 1,000 PY in 2014. In women, nitrofuran derivatives (J01XE) were clearly the most frequently prescribed drugs since 1999, with a strong and significant increase in the last years from 52 prescriptions per 1,000 PY in 2008 to 98 prescriptions per 1,000 PY in 2014. They increasingly replaced the prescriptions of trimethoprim and derivatives (J01EA) and fluoroquinolones (J01MA), which were also commonly prescribed in women. Also, the prescriptions of combinations of penicillins, incl. beta-lactamase inhibitors (J01CR) and fosfomycin significantly increased, mainly in most recent years (**Figure 2**).

Adherence to guidelines

The implementation of the Dutch guideline on the treatment of UTI of 2005 was associated in women with a significant decrease of the slope of prescriptions of trimethoprim and derivatives (J01EA) and an increase in nitrofuran derivatives (J01XE) prescriptions. Unfortunately, we did not have sufficient data before 1999 and after 2013 to study the implementation of the other guidelines. For men, no significant effects were found of the implementation of the guideline on the slope of any prescriptions.

Prescriptions of antimicrobial drugs for UTIs by age groups

For peer review only

For peer review only - http://bmjopen.bmj.com/site/about/guidelines.xhtml

Both in men and women, an increase in total number of UTI prescriptions was observed by age category. In women the number of prescriptions in all age groups is higher than in men, with nitrofuran derivatives (J01XE) as the most prescribed antimicrobial drug in all age groups, followed by fluoroquinolones (J01MA) and trimethoprim and derivatives (J01EA). In men, fluoroquinolones (J01MA) are the most frequently prescribed antimicrobial drugs in most age groups, followed by nitrofuran derivatives (J01XE) and combinations of penicillins, incl. beta-lactamase inhibitors (J01CR) **(Figure 3)**.

Analyzing the total number of prescriptions for UTIs (J01 – antibacterials for systemic use) per age category, we observed an increase in the last years for all age categories, but most prominent in the higher age categories **(Figure S3)**.

Changes in duration of nitrofurantoin prescriptions

In total, 215,531 prescriptions of nitrofurantoin with an ICPC code for UTIs were prescribed to women in the study period. In line with a change in recommended duration from 3 to 5 days in 2005, the proportion of prescriptions with a duration of 5 days increased strongly from 2005 onwards, while proportion of prescriptions with a duration of 3 days became scarce. The proportion of prescriptions with a duration of 7 days decreased from 33% in 1996 to 18% in 2014. The proportion of prescriptions with a duration other than 3, 5 or 7 days (defined as "other duration") remained stable over time, with a proportion ranging between 6% and 10% **(Figure 4)**.

For peer review only - http://bmjopen.bmj.com/site/about/guidelines.xhtml

For peer review only

Discussion

In this study we investigated the prescribing of antimicrobial drugs for UTIs in primary care during the study period January 1st 1996 until January 1st 2015. To assess compliance to Dutch guidelines on the treatment of UTI in primary care, the choice of antimicrobial drug was compared to the recommendations in these guidelines. In men, the number of nitrofurantoin prescriptions increased, but fluoroquinolones were the most commonly prescribed antimicrobial drug in all years, although they are only recommended as first-choice option in the most recent guideline. Women were most often prescribed nitrofuran derivatives (J01XE), which increased over the years, followed by trimethoprim and derivatives (J01EA) and fluoroquinolones (J01MA), which both decreased. Furthermore, compliance with respect to the duration of nitrofurantoin use in women has increased since the guideline of 2005.

Nitrofurantoin was shown to be the most prescribed drugs for the treatment of UTIs, especially in women, and increasingly in men. The Dutch guidelines of 1989 indicated nitrofurantoin as one of the options for women, whereas it was suggested as option for both men and women in the guideline of 1999 and as first-choice drug for cystitis in the 2005 and 2013 guideline. This is reflected in our findings; we observed in men and women an increase in nitrofurantoin prescriptions since 1999, with a steep and significant increase since 2005. In this period nitrofurantoin replaced trimethoprim in women, which significantly decreased after 2005. In the guidelines of 1999 and 2005, amoxicillin-clavulanic acid was recommended for UTIs with tissue invasion, whereas amoxicillin (or trimethoprim in men) was recommended for UTIs with tissue invasion in the guidelines prior to 1999. In men and women, a significant increase of combinations of penicillins, incl. beta-lactamase inhibitors such as amoxicillin-clavulanic acid, was shown during the study period. In men, a flattening was seen from 2012 on, just before the guideline of 2013, which recommended ciprofloxacin as first-choice.

For peer review only - http://bmjopen.bmj.com/site/about/guidelines.xhtml

A deviation from the UTI guidelines was observed with regard to the prescribing of fluoroquinolones. Fluoroquinolones, in particular ciprofloxacin, were only advised as first-choice drug for UTIs with tissue invasion from 2013 on (in 2005 it was 2nd choice and in 1989 use was discouraged), whereas in men these drugs were the most frequently prescribed antimicrobial drug during the complete study period. In women, we saw a significant decrease over time suggesting an increase of compliance of GPs over time. We also studied the adherence to guidelines with regard to the duration of nitrofurantoin prescribing in women. The recommended duration increased from 3 to 5 days in 2005, which is reflected in our findings, indicating an increase of adherence to the guidelines by GPs, mainly since the guidelines of 2005 and 2013.[4-7]

A study in another Dutch GP database investigated antimicrobial prescribing for several infections during 2001 and showed that approximately 75% of the prescriptions for cystitis were first-choice drugs (nitrofurantoin or trimethoprim) according to guidelines.[13] The percentage of first-choice drug prescriptions in our study in 2001 is comparable (approximately 67% for women). A smaller study from 2009 in 970 non-pregnant women of 11 years and older with cystitis determined that 66% were prescribed nitrofurantoin, whereas we found a proportion of 57% in all women in 2009.[14] Our proportion is somewhat smaller, possibly because we did not distinguish between cystitis and pyelonephritis, since a proportion of the prescriptions had an indication code for both. However, the sample size of the other study is much smaller resulting in more uncertainty around the true prevalence of antimicrobial drug use.

As for all observational studies, our study has strengths and limitations. The strength is the use of a large population-based cohort from a database with detailed information on prescriptions and where information is collected as part of routine clinical care reducing the risk of selection and information bias. However, as part of new health quality standards, GPs are encouraged to improve disease coding, a

For peer review only - http://bmjopen.bmj.com/site/about/guidelines.xhtml

phenomenon also observed in other countries. This might in part explain the increase of total prescriptions for UTIs which is underlined by our finding that the number of prescriptions for nitrofurantoin without an indication code seemed to decrease, especially in women since approximately 2007. To control for this bias, we analyzed UTI prescriptions as a proportion of the total number of antimicrobial drug prescriptions with an indication code (thus excluding prescriptions without a disease code). Yet, we observed that the increase of prescriptions was larger for UTI's than for other indications. This increase in antimicrobial drug prescribing for the treatment of UTI is supported by other literature. According to the NIVEL (Netherlands institute for health services research) registration, the number of contacts with a GP for UTIs increased (especially in women) from 185 per 1,000 in 2012 to 295 per 1,000 in 2015.[3] This was confirmed by another large Dutch GP database, showing an increase of GP visits for UTIs and AB prescriptions for UTI in the period 2007-2010.[15 16] This increase of use of antimicrobial drugs for UTIs might partly be explained by ageing of the population. Indeed, we showed that antimicrobial drugs were especially prescribed to patients in older age categories. Additionally, a Dutch study from 2012 also showed an increase of antimicrobial drug prescribing by age, and an increase of total prescriptions of antimicrobial drugs to elderly: in 2000, 9% of patients above 80 years had at least one prescription, which increased to 22% in 2009.[8] However, ageing of the population might not solely explain the increase, since we also showed an increase in prescriptions of antimicrobial drugs in younger age groups. Possible explanations might be that more patients suffer from recurrent UTIs, that patients more frequently visit their GP in case of a UTI or that GPs more easily prescribe antimicrobial drugs for UTIs, but this has to be elucidated in future studies.

Reducing the use of antimicrobial drugs is important, since the drawback of treating infections with antimicrobial drugs is the development of AMR.[17] Patients suffering from infections caused by resistant bacteria have an increased risk of a worse clinical outcome and death.[17] Moreover, AMR leads to an increase in healthcare costs, which has been estimated to be 1.5 billion euros in EU every year.[18 19]

For peer review only - http://bmjopen.bmj.com/site/about/guidelines.xhtml

Misuse and overuse of antimicrobial drugs are the most important risk factors of antimicrobial resistance worldwide.[20] Therefore, antimicrobial stewardship programs have been developed, aiming to optimize the prescribing of antimicrobial drugs, minimize misuse and overuse in order to minimize AMR.[21] Our study demonstrates that databases with antimicrobial drug prescriptions and indications are useful for surveillance of antimicrobial drug prescriptions over time. In future, information gathered in such databases should be provided to GP practices as part of antibiotic stewardship in primary care.

Funding

The authors have no funding to report.

Competing interests statement

KV works for a department who in the past received unconditional grants from: Pfizer/BI, Yamanouchi, GSK, Novartis. None of which are related to the content of this paper. The other authors have nothing to declare.

Author's contributions

MM, AV, BS and KV designed the study. MM, EB and KV analyzed the data and interpreted these with the aid of AV and BS. MM wrote the manuscript with critical input from all co-authors.

Data sharing statement

The aggregated data are available with the corresponding author upon reasonable request.

Patients and public involvement

No patients were involved in any stage of this study.

For peer review only

For peer review only - http://bmjopen.bmj.com/site/about/guidelines.xhtml

References

1. Foxman B. Epidemiology of urinary tract infections: incidence, morbidity, and economic costs. Am J Med 2002;**113 Suppl 1A**:5S-13S.
2. Foxman B. The epidemiology of urinary tract infection. Nat Rev Urol 2010;**7**(12):653-60.
3. NIVEL Zorgregistraties eerste lijn 2015: Nederlands instituut voor onderzoek van de gezondheidszorg; [updated 02-02-2016. Available from: https://www.nivel.nl/nl/NZR/huisarts-top-20-diagnoses-bij-contacten-naar-geslacht.
4. van Pinxteren BK, B. J.; Geerlings, S. E.; Visser, H. S., Klinkhamer, S.; van der Weele, G. M.; Verduijn, M. M.; Opstelten, W.; Burgers, J. S.; van Asselt, K. M. NHG-Standaard Urineweginfecties. Huisarts en Wetenschap 2013;**56**(6):270-80.
5. Timmermans AEB, P. J. A. M.; Winkens, R. A. G.; Arets, H.; Wiersma, T. . NHG-Standaard Urineweginfecties. Huisarts en Wetenschap 1999;**42**(13):613-22.
6. van Balen FAMB, P. J. A. M.; van Pienbroek, E.; Winkens, R.A.G. NHG-Standaard Urineweginfecties Huisarts en Wetenschap 1989;**32**(11):439-43.
7. van Haaren KAMV, H. S.; van Vliet, S.; Timmermans, A. E.; Yadava, R.; Geerlings, S. E.; ter Riet, G.; van Pinxteren, B. NHG-Standaard Urineweginfecties Huisarts en Wetenschap 2005;**48**(7):341-52.
8. Haeseker MB, Dukers-Muijrers NH, Hoebe CJ, et al. Trends in antibiotic prescribing in adults in Dutch general practice. PLoS One 2012;**7**(12):e51860.
9. NethMap. Consumption of antimicrobial agents and antimicrobial resistance among medically important bacteria in The Netherlands in 2015: SWAB/RIVM, 2016.
10. Vlug AE, van der Lei J, Mosseveld BM, et al. Postmarketing surveillance based on electronic patient records: the IPCI project. Methods Inf Med 1999;**38**(4-5):339-44.
11. WCCfDSM. WHO Collaborating Centre for Drug Statistics Methodology; [Available from: https://www.whocc.no/.
12. (EPOC) CEPaOoC. Interrupted time series (ITS) analyses: EPOC Resources for review authors; 2017 [Available from: http://epoc.cochrane.org/epoc-specific-resources-review-authors.
13. Ong DS, Kuyvenhoven MM, van Dijk L, et al. Antibiotics for respiratory, ear and urinary tract disorders and consistency among GPs. J Antimicrob Chemother 2008;**62**(3):587-92.
14. den Heijer DD, GA. Maes, J. Stobberingh, EE. Antibiotica bij ongecompliceerde urineweginfecties: geen toename van resistentie in de afgelopen 5 jaar. Nederlands Tijdschrift voor Geneeskunde 2011;**155**.
15. Willems CS, van den Broek D'Obrenan J, Numans ME, et al. Cystitis: antibiotic prescribing, consultation, attitudes and opinions. Fam Pract 2014;**31**(2):149-55.
16. van den Broek d'Obrenan J, Verheij TJ, Numans ME, et al. Antibiotic use in Dutch primary care: relation between diagnosis, consultation and treatment. J Antimicrob Chemother 2014;**69**(6):1701-7.
17. Bergman M, Nyberg ST, Huovinen P, et al. Association between antimicrobial consumption and resistance in Escherichia coli. Antimicrob Agents Chemother 2009;**53**(3):912-7.
18. Cosgrove SE. The relationship between antimicrobial resistance and patient outcomes: mortality, length of hospital stay, and health care costs. Clin Infect Dis 2006;**42 Suppl 2**:S82-9.
19. The bacterial challenge: time to react. A call to narrow the gap between multidrug-resistant bacteria in the EU and the development of new antibacterial agents: ECDC/EMEA Joint technical report, 2009.
20. Ventola CL. The antibiotic resistance crisis: part 1: causes and threats. P T 2015;**40**(4):277-83.
21. Ventola CL. The antibiotic resistance crisis: part 2: management strategies and new agents. P T 2015;**40**(5):344-52.

For peer review only - http://bmjopen.bmj.com/site/about/guidelines.xhtml

Figure legends

Figure 1: The number of users and prescriptions of antimicrobial drugs for UTIs, other infections and infections without indication code in the period 1996 – 2014. Users show the number of patients who were prescribed at least 1 antimicrobial drug of the drug group of interest in that specific year per 1,000 PY. Prescriptions show the total number of prescriptions who were prescribed in that specific year per 1,000 PY. Other infections included all users/prescriptions for infections with an indication code other than UTIs, such as respiratory infections, ear infections and skin infections.

Figure 2: The number of users and prescriptions of antimicrobial drugs for urinary tract infections in the period 1996 – 2014. Users men and women show the number of patients who were prescribed at least 1 antimicrobial drug of the drug group of interest in that specific year per 1,000 PY. Prescriptions men and women show the total number of prescriptions who were prescribed in that specific year per 1,000 PY. The figure only shows the most prevalent drug groups J01CA (penicillins with extended spectrum), J01CR (combinations of penicillins, incl. beta-lactamase inhibitors), J01EA (trimethoprim and derivatives), J01EE (combinations of sulfonamides and trimethoprim, incl. derivatives), J01MA (fluoroquinolones), J01XE (nitrofuran derivatives), J01XX (other antimicrobial drugs). The prevalence of other antimicrobial drug classes was analyzed but was because of low numbers not described in the results section. Note that the scale of the y-axis differs between men and women.

Figure 3: The number of users and prescriptions of antimicrobial drugs for urinary tract infections per age group. Users men and women show the number of patients who got prescribed at least 1 antimicrobial drug of the drug group in that specific age group during the total study time per 1,000 PY. Prescriptions men and women show the total number of prescriptions that were prescribed in that specific age group during the total study time per 1,000 PY. The figure only shows the most prevalent drug groups J01CA (penicillins with extended spectrum), J01CR (combinations of penicillins, incl. beta-lactamase inhibitors), J01EA (trimethoprim and derivatives), J01EE (combinations of sulfonamides and trimethoprim, incl. derivatives), J01MA (fluoroquinolones), J01XE (nitrofuran derivatives), J01XX (other antimicrobial drugs). Note that the scale of the y-axis differs between men and women.

Figure 4: The proportion of the different durations of nitrofurantoin prescriptions in women in the period 1996 – 2014. The proportion of prescriptions of nitrofurantoin prescriptions (J01XXE01) with a duration of 3, 5, 7 days or "other" duration (unequal to 3, 5, 7 days or unknown) with in de denominator all these prescriptions. Until 2005 the duration recommended by the guideline was 3 days, whereas since guideline of 2005 the advised duration was 5 days.

For peer review only - http://bmjopen.bmj.com/site/about/guidelines.xhtml

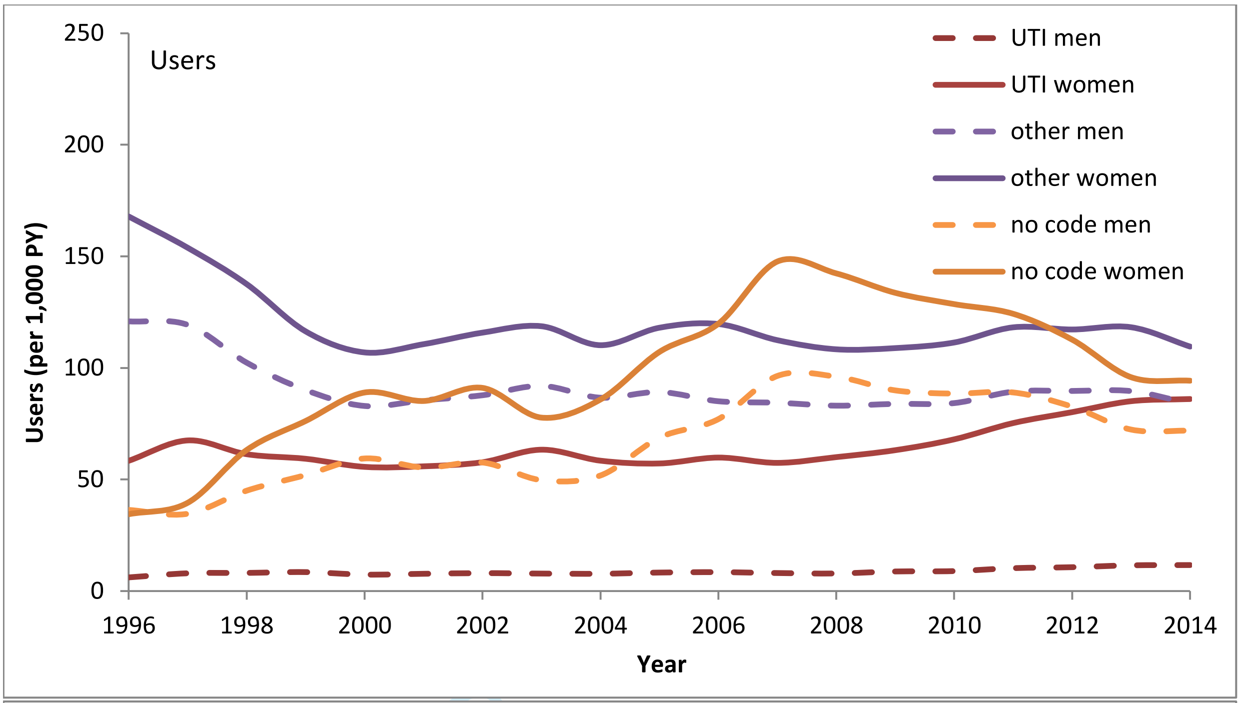
Users
Users (per 1,000 PY)
Year
UTI men
UTI women
other men
other women
no code men
no code women

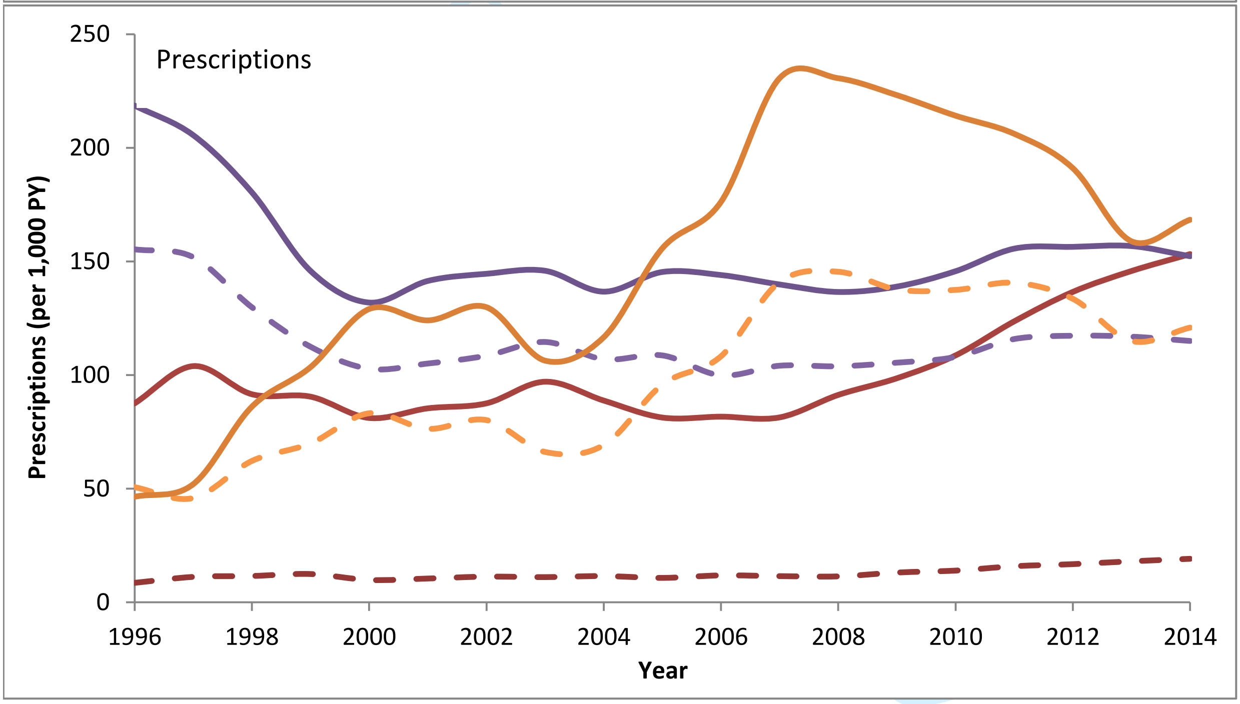
Prescriptions
Prescriptions (per 1,000 PY)
Year

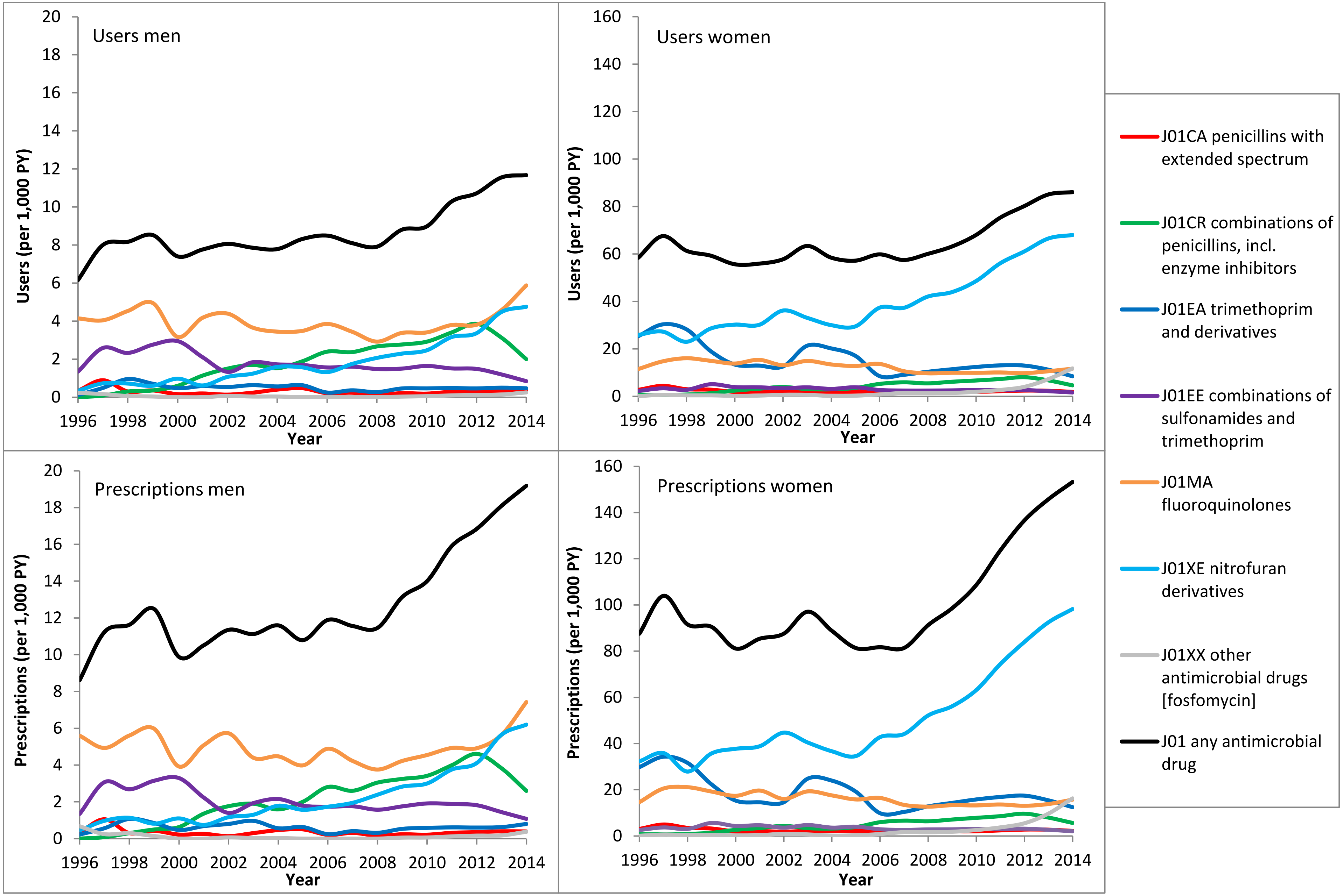
Users men
Users (per 1,000 PY)
Year
Users women
Users (per 1,000 PY)
Year
Prescriptions men
Prescriptions (per 1,000 PY)
Year
Prescriptions women
Prescriptions (per 1,000 PY)
Year
J01CA penicillins with extended spectrum
J01CR combinations of penicillins, incl. enzyme inhibitors
J01EA trimethoprim and derivatives
J01EE combinations of sulfonamides and trimethoprim
J01MA fluoroquinolones
J01XE nitrofuran derivatives
J01XX other antimicrobial drugs [fosfomycin]
J01 any antimicrobial drug

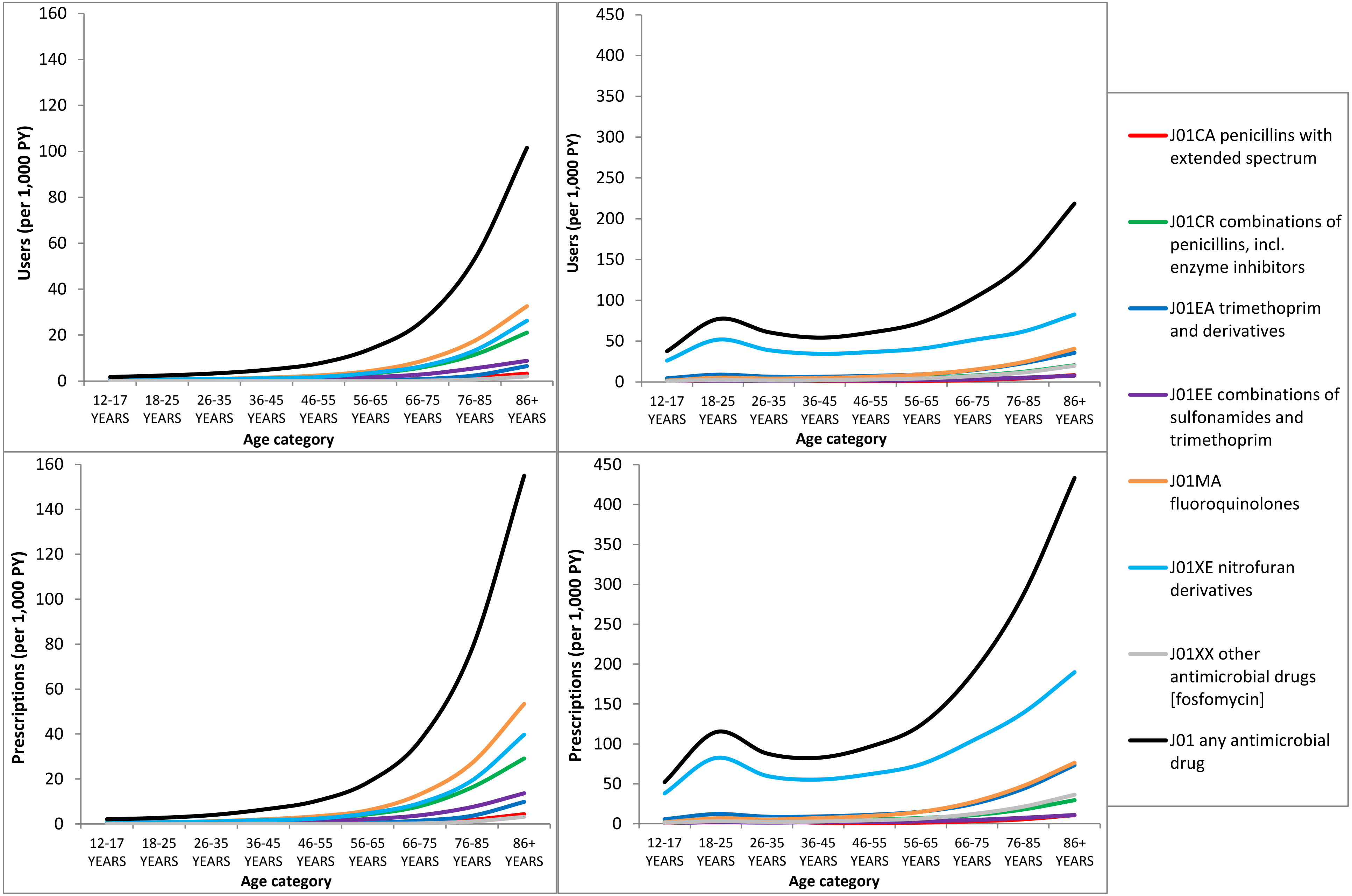
160
140
120
100
80
60
40
20
0
Users (per 1,000 PY)
12-17 YEARS
18-25 YEARS
26-35 YEARS
36-45 YEARS
46-55 YEARS
56-65 YEARS
66-75 YEARS
76-85 YEARS
86+ YEARS
Age category
450
400
350
300
250
200
150
100
50
0
Users (per 1,000 PY)
Age category
Prescriptions (per 1,000 PY)
Age category
Prescriptions (per 1,000 PY)
Age category
J01CA penicillins with extended spectrum
J01CR combinations of penicillins, incl. enzyme inhibitors
J01EA trimethoprim and derivatives
J01EE combinations of sulfonamides and trimethoprim
J01MA fluoroquinolones
J01XE nitrofuran derivatives
J01XX other antimicrobial drugs [fosfomycin]
J01 any antimicrobial drug

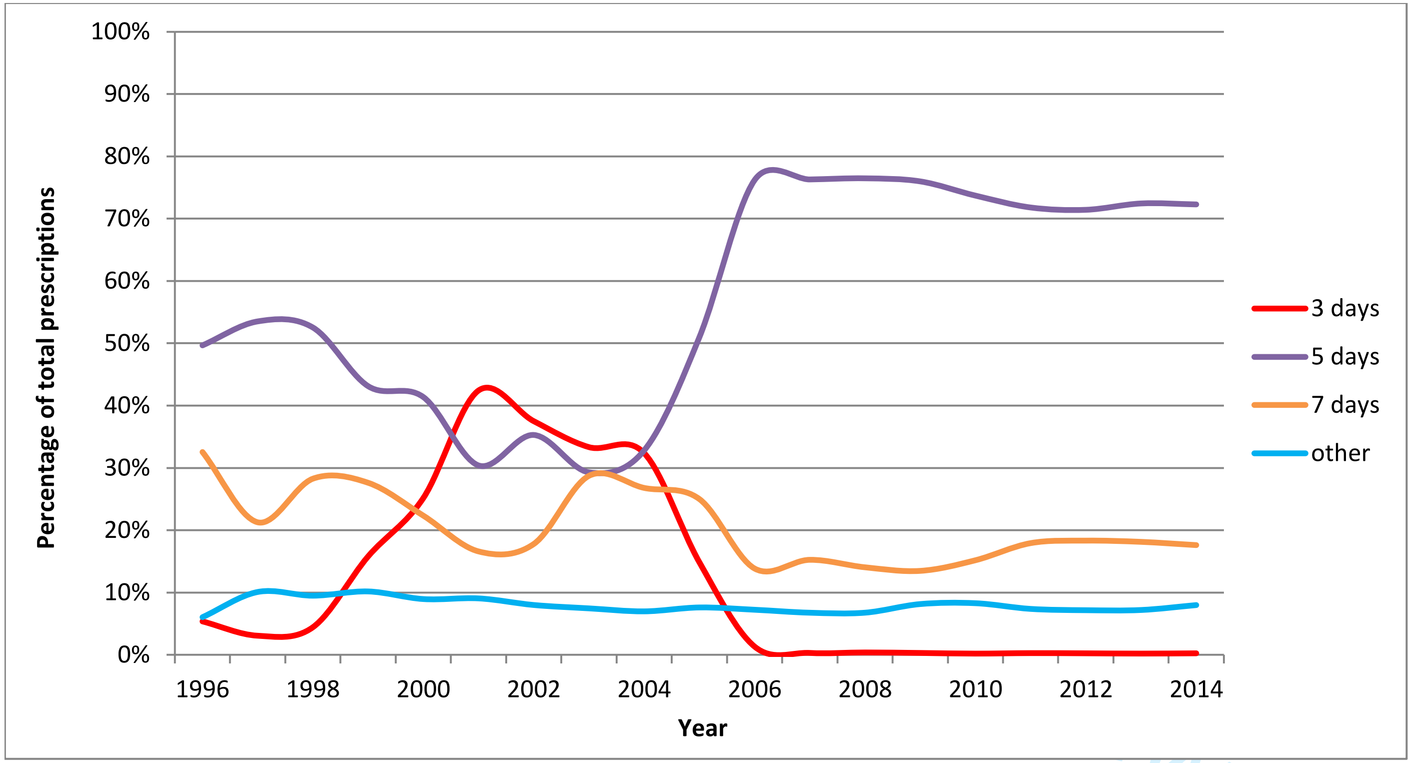

For peer review only - http://bmjopen.bmj.com/site/about/guidelines.xhtml

Supplementary table 1: Antimicrobial drug classes

ATC code	Drug class
J01AA*	Tetracyclines
J01CA	Penicillins with extended spectrum
J01CE*	Beta-lactamase sensitive penicillins
J01CF*	Beta-lactamase resistant penicillins
J01CR	Combinations of penicillins, incl. beta-lactamase inhibitors
J01DB*	First-generations cephalosporins
J01DC*	Second-generation cephalosporins
J01DD*	Third-generation cephalosporins
J01EA	Trimethoprim and derivatives
J01EB*	Short-acting sulfonamides
J01EE	Combinations of sulfonamides, and trimethoprim, incl. derivatives
J01FA*	Macrolides
J01FF*	Lincosamides
J01GB*	Other aminoglycosides
J01MA	Fluoroquinolones
J01MB*	Other quinolones
J01XC*	Steroid antibacterials
J01XE	Nitrofuran derivatives
J01XX	Other antibacterials

All antimicrobial drug classes that were prescribed for UTIs in the study population. The number of prescriptions of drugs with * was very low and are not shown in the figures.

For peer review only

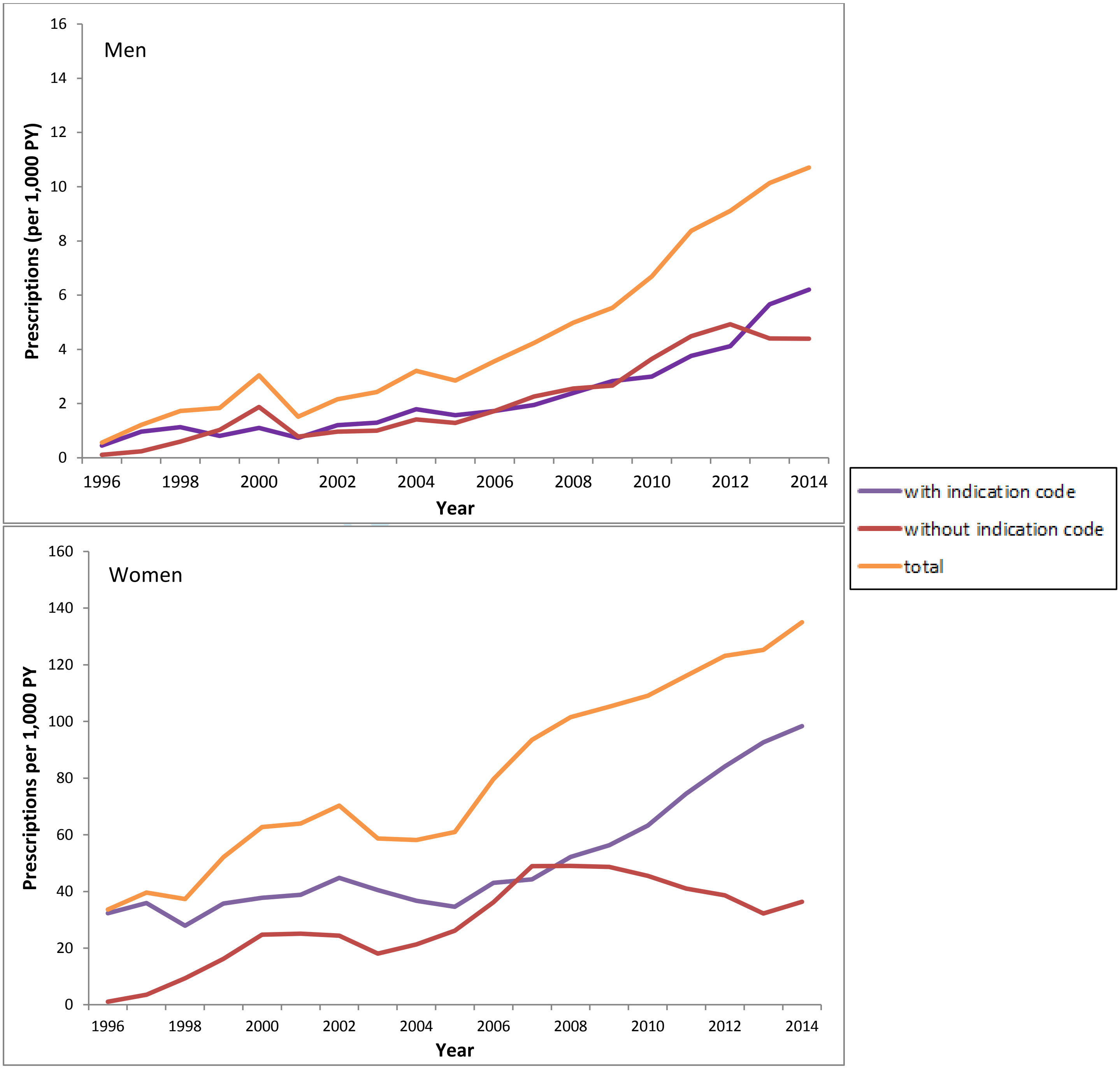

Supplementary figure 1: The number of prescriptions of nitrofurantoin with and without an indication code for urinary tract infections for men and women. Note that the scale of the y-axis differs between men and women.

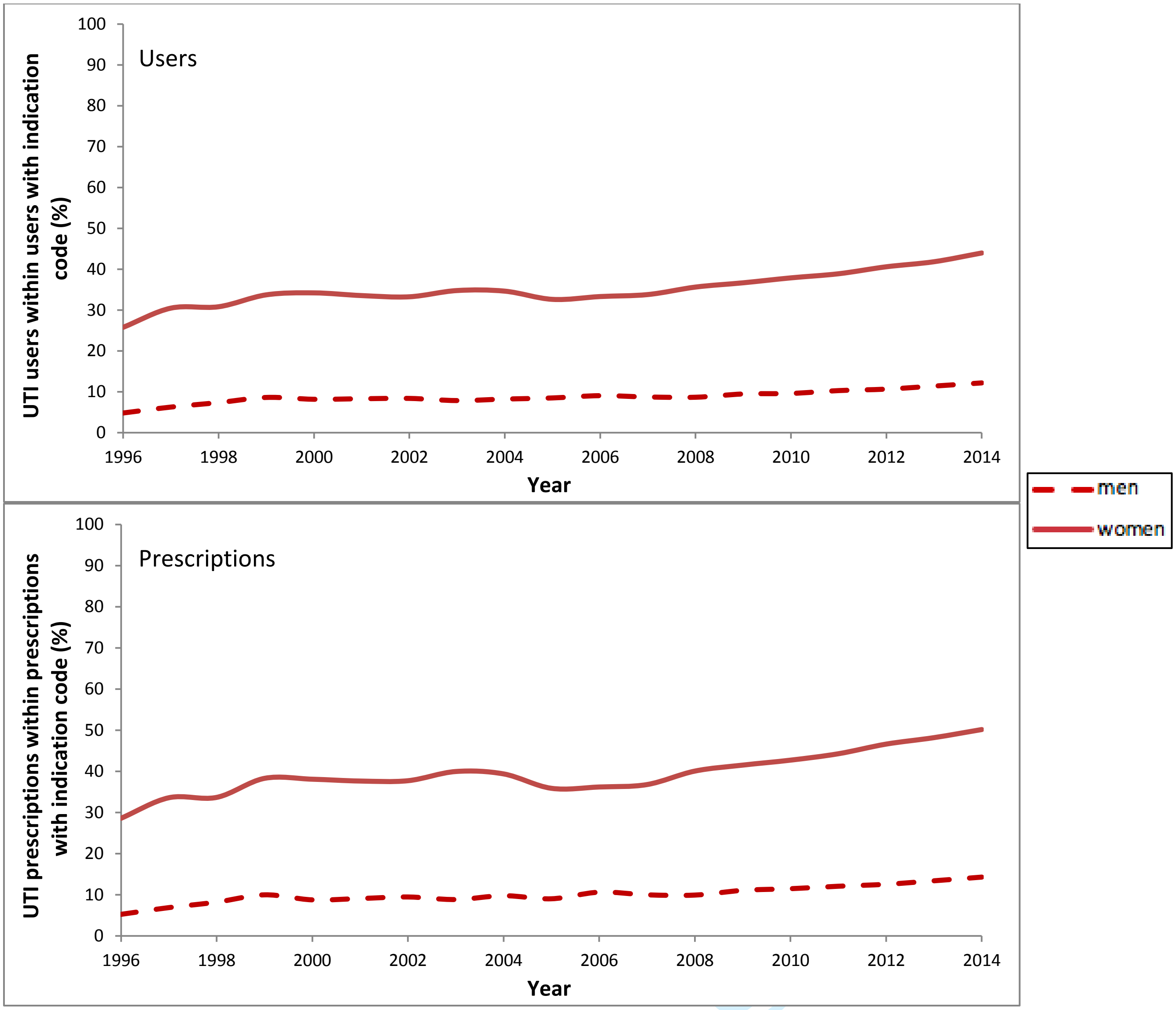

Supplementary figure 2: The percentage of users/prescriptions with an indication code for UTIs within all users/prescriptions with an indication code (thus including indication codes for UTIs, airway infections, ear infections, skin infections etc.).

For peer review only - http://bmjopen.bmj.com/site/about/guidelines.xhtml

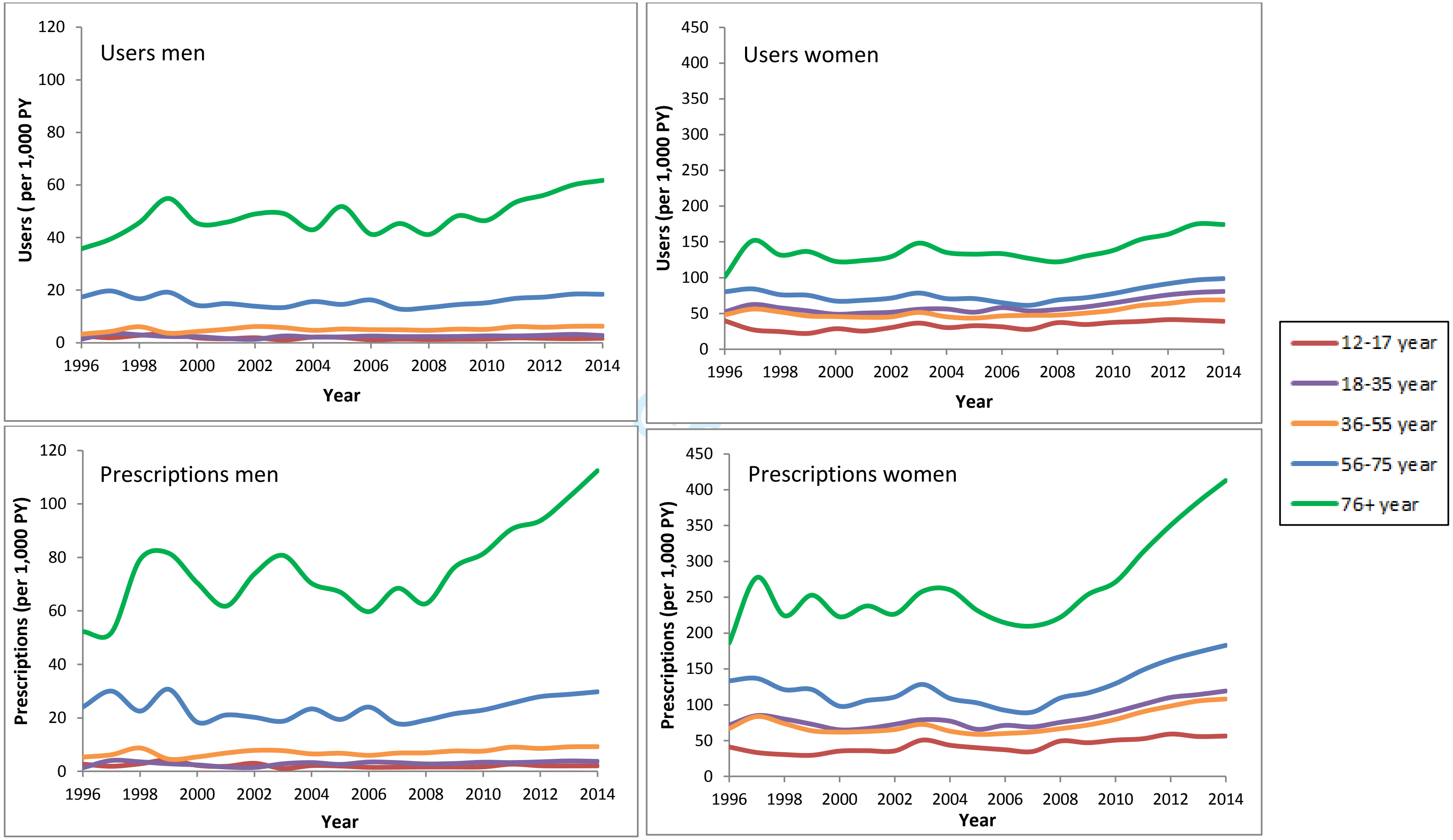

Supplementary figure 3: The number of users and prescriptions of antimicrobial drugs for urinary tract infections for different age groups per year. Users men and **women** show the number of patients who got prescribed at least 1 antimicrobial drug in that specific age group per year per 1,000 PY. **Prescriptions men** and **women** show the total number of antimicrobial drug prescriptions that were prescribed in that specific age group per year per 1,000 PY. Note that the scale of the y-axis differs between men and women.

For peer review only

STROBE Statement—checklist of items that should be included in reports of ~~o~~bservational studies

Trends of prescribing antimicrobials drugs for UTIs in primary care in the Netherlands

	Item No	Recommendation	
**Title and abstract**	1	(*a*) Indicate the study's design with a commonly used term in the title or the abstract	✓ p1
		(*b*) Provide in the abstract an informative and balanced summary of what was done and what was found	✓ p2
**Introduction**			
Background/rationale	2	Explain the scientific background and rationale for the investigation being reported	✓ p4
Objectives	3	State specific objectives, including any prespecified hypotheses	✓ p4
**Methods**			
Study design	4	Present key elements of study design early in the paper	✓ p6
Setting	5	Describe the setting, locations, and relevant dates, including periods of recruitment, exposure, follow-up, and data collection	✓ p6/p7
Participants	6	(*a*) *Cohort study*—Give the eligibility criteria, and the sources and methods of selection of participants. Describe methods of follow-up<br>*Case-control study*—Give the eligibility criteria, and the sources and methods of case ascertainment and control selection. Give the rationale for the choice of cases and controls<br>*Cross-sectional study*—Give the eligibility criteria, and the sources and methods of selection of participants	✓ p6/p7
		(*b*) *Cohort study*—For matched studies, give matching criteria and number of exposed and unexposed<br>*Case-control study*—For matched studies, give matching criteria and the number of controls per case	NA
Variables	7	Clearly define all outcomes, exposures, predictors, potential confounders, and effect modifiers. Give diagnostic criteria, if applicable	✓ p6-8
Data sources/ measurement	8*	For each variable of interest, give sources of data and details of methods of assessment (measurement). Describe comparability of assessment methods if there is more than one group	✓ p6-8
Bias	9	Describe any efforts to address potential sources of bias	✓ p6-8 ✓ p9-10
Study size	10	Explain how the study size was arrived at	✓ p6/7
Quantitative variables	11	Explain how quantitative variables were handled in the analyses. If applicable, describe which groupings were chosen and why	✓ p6-8
Statistical methods	12	(*a*) Describe all statistical methods, including those used to control for confounding	✓ p8
		(*b*) Describe any methods used to examine subgroups and interactions	
		(*c*) Explain how missing data were addressed	
		(*d*) *Cohort study*—If applicable, explain how loss to follow-up was addressed<br>*Case-control study*—If applicable, explain how matching of cases and controls was addressed<br>*Cross-sectional study*—If applicable, describe analytical methods taking account of sampling strategy	
		(*e*) Describe any sensitivity analyses	

Continued on next page

**Results**			
Participants	13*	(a) Report numbers of individuals at each stage of study—eg numbers potentially eligible, examined for eligibility, confirmed eligible, included in the study, completing follow-up, and analysed	✓ P9
		(b) Give reasons for non-participation at each stage	
		(c) Consider use of a flow diagram	
Descriptive data	14*	(a) Give characteristics of study participants (eg demographic, clinical, social) and information on exposures and potential confounders	✓ P9
		(b) Indicate number of participants with missing data for each variable of interest	
		(c) *Cohort study*—Summarise follow-up time (eg, average and total amount)	✓ P9
Outcome data	15*	*Cohort study*—Report numbers of outcome events or summary measures over time	✓ P9
		Case-control study—Report numbers in each exposure category, or summary measures of exposure	
		Cross-sectional study—Report numbers of outcome events or summary measures	
Main results	16	(*a*) Give unadjusted estimates and, if applicable, confounder-adjusted estimates and their precision (eg, 95% confidence interval). Make clear which confounders were adjusted for and why they were included	NA
		(*b*) Report category boundaries when continuous variables were categorized	P9-11
		(*c*) If relevant, consider translating estimates of relative risk into absolute risk for a meaningful time period	
Other analyses	17	Report other analyses done—eg analyses of subgroups and interactions, and sensitivity analyses	✓ P9-11
**Discussion**			
Key results	18	Summarise key results with reference to study objectives	✓ P12
Limitations	19	Discuss limitations of the study, taking into account sources of potential bias or imprecision. Discuss both direction and magnitude of any potential bias	✓ P13/14
Interpretation	20	Give a cautious overall interpretation of results considering objectives, limitations, multiplicity of analyses, results from similar studies, and other relevant evidence	✓ P12-14
Generalisability	21	Discuss the generalisability (external validity) of the study results	✓ P15
**Other information**			
Funding	22	Give the source of funding and the role of the funders for the present study and, if applicable, for the original study on which the present article is based	✓ P15

*Give information separately for cases and controls in case-control studies and, if applicable, for exposed and unexposed groups in cohort and cross-sectional studies.

Note: An Explanation and Elaboration article discusses each checklist item and gives methodological background and published examples of transparent reporting. The STROBE checklist is best used in conjunction with this article (freely available on the Web sites of PLoS Medicine at http://www.plosmedicine.org/, Annals of Internal Medicine at http://www.annals.org/, and Epidemiology at http://www.epidem.com/). Information on the STROBE Initiative is available at www.strobe-statement.org.

BMJ Open

Trends of prescribing antimicrobial drugs for urinary tract infections in primary care in the Netherlands: a population-based cohort study

Journal:	*BMJ Open*
Manuscript ID	bmjopen-2018-027221.R1
Article Type:	Research
Date Submitted by the Author:	13-Feb-2019
Complete List of Authors:	Mulder, Marlies; Erasmus MC, Department of Epidemiology; Health and Youth Care Inspectorate<br>Baan, Esme; Erasmus MC, Department of Medical Informatics<br>Verbon, Annelies ; Erasmus MC, Medical Microbiology and Infectious diseases<br>Stricker, Bruno; Ersamus MC, Department of Epidemiology; Health and Youth Care Inspectorate<br>Verhamme, Katia; Erasmus Medical Center, Department of Medical Informatics; Universiteit Gent, Department of Bioanalysis
**Primary Subject Heading**:	Pharmacology and therapeutics
Secondary Subject Heading:	Infectious diseases, Epidemiology, General practice / Family practice
Keywords:	Urinary tract infections < UROLOGY, EPIDEMIOLOGY, PRIMARY CARE, BACTERIOLOGY, Quality in health care < HEALTH SERVICES ADMINISTRATION & MANAGEMENT

SCHOLARONE™
Manuscripts

Trends of prescribing antimicrobial drugs for urinary tract infections in primary care in the Netherlands: a population-based cohort study

Running title: Prescribing of antimicrobial drugs for UTIs by GPs in the Netherlands

M. Mulder[1,2], E.J. Baan[3], A. Verbon[4], B.H.C. Stricker[1,2,5], K.M.C. Verhamme[3,6]

1. Department of Epidemiology, Erasmus Medical Centre, PO Box 2040, 3000 CA Rotterdam, The Netherlands

2. Health and Youth Care Inspectorate, Postbus 2518, 6401 DA Heerlen, The Netherlands

3. Department of Medical Informatics, Erasmus Medical Centre, PO Box 2040, 3000 CA Rotterdam, The Netherlands

4. Department of Medical Microbiology and Infectious Diseases, Erasmus Medical Centre, PO Box 2040, 3000 CA Rotterdam, The Netherlands

5. Department of Internal Medicine, Erasmus Medical Centre, PO Box 2040, 3000 CA Rotterdam, The Netherlands

6. Department of Bioanalysis, Faculty of Pharmaceutical Sciences, University of Ghent, Belgium

Keywords: antibiotic stewardship, antimicrobial drug prescribing, urinary tract infections, primary care.

Address for Correspondence:
Professor Bruno H.Ch. Stricker
Department of Epidemiology
Erasmus MC - University Medical Center Rotterdam
P.O. Box 2040
3000 CA Rotterdam
The Netherlands
b.stricker@erasmusmc.nl

For peer review only

Abstract

Objective: Urinary tract infections (UTIs) are an important reason to consult a General Practitioner (GP) Here, we describe antimicrobial drug prescribing patterns for UTIs by GPs in relation to the Dutch primary care guidelines.

Methods: We conducted a population-based cohort study in the Dutch Integrated Primary Care Information database (IPCI), which encompasses approximately 2.5 million patients. All patients ≥12 years with at least one year of follow-up from 1996 to 2014 were extracted from the database. The number of prescriptions and choice of drug type were investigated over time and in different age categories. The choice of antimicrobial drugs classes for UTIs and the duration of nitrofurantoin use in women were compared to the Dutch primary care guidelines of 1989, 1999, 2005 and 2013.

Results: The source population comprised 1,755,085 patients who received 2,019,335 antimicrobial drug prescriptions; 401,655 (35.1%) prescriptions were for UTIs (45.2% in women; 12.6% in men). The proportion of prescriptions for UTI within all prescriptions with an indication code increased from 5.2% in 1996 to 14% in 2014 in men and from 28% in 1996 to 50% in 2014 for women. In men, UTIs were most frequently treated with fluoroquinolones during the entire study period, whereas fluoroquinolones were only advised as first-choice in the latest guideline of 2013. In women, UTIs were increasingly ($p<0.05$) treated with nitrofuran derivatives with a statistically significant difference after implementation of the guideline of 2005. Compliance to the advised duration of nitrofurantoin prescriptions in women has increased since the guideline of 2005.

Conclusions: The antimicrobial drug prescribing for UTI seemed to have increased over time. Prescribing in line to the UTI guidelines increased with regards to choice and duration of antimicrobial drugs. We showed that databases like IPCI in which prescription and indication are monitored, can be valuable antibiotic stewardship tools.

For peer review only

Strengths and limitations of this study

- This study was conducted in a large database, including a substantial part (~15%) of the Dutch population, implying that the external validity of our study is high.
- Every individual in the Netherlands is assigned to a single GP and antimicrobial drugs are not sold over the counter. This limits information bias, since we had access to all antimicrobial drug prescriptions prescribed in primary care.
- Improvement of coding discipline in more recent years might bias conclusions on total prescribing of antimicrobial drugs, but we assume that this bias would be similar for all indications. Furthermore, we compared the antimicrobial drug prescription data for UTIs with antimicrobial drug prescription data for other infections.
- We analyzed the choice of antimicrobial drugs prescribed by GPs in relation to the Dutch guidelines on treatment of urinary tract infections and show that database studies can be used as a tool for good antimicrobial stewardship.

For peer review only

Introduction

Urinary tract infections (UTIs) are among the most common infections in humans. UTIs cause a substantial burden of disease with major economic consequences.[1 2] In women, UTIs are the most common reason to consult a general practitioner (GP) in the Netherlands with 232 contacts per 1,000 women in 2014. In men, this number was substantially lower: 37 contacts per 1,000 men.[3]

In most cases UTIs are treated with antimicrobial drugs. The choice of antimicrobial drug depends on the antimicrobial sensitivity of pathogens in urinary cultures (if taken), severity of the symptoms and potential co-morbidities of the individual. Based on the resistance patterns of *E. coli* in the Netherlands, Dutch guidelines on the treatment of UTI have changed several times in the last few decades. The latest Dutch guideline for the treatment of urinary UTIs in primary care was released in 2013 and recommends nitrofurantoin as first-choice in all patients with cystitis, including men. In case of signs of tissue invasion, ciprofloxacin is recommended as first-choice.[4] Additionally, the recommended duration of treatment has changed. The recommended duration of treatment with nitrofurantoin for cystitis in women was 3 days in the guidelines of 1989 and 1999, whereas it has been extended to 5 days in the guidelines of 2005 and 2013 (**Table 1**).[4-7]

Table 1: Overview of the treatment of UTIs according to the Dutch guidelines for GPs

	1989	1999	2005	2013
**Women**				
Cystitis	Trimethoprim (J01EA) *or* Sulfamethizol (J01EB) *or* Nitrofurantoin (J01XE) ***3 days***	Nitrofurantoin (J01XE) ***3 days*** *or* Trimethoprim (J01EA)	*1st choice*: Nitrofurantoin (J01XE) ***5 days*** *2nd choice*: Trimethoprim (J01EA) *3rd choice*: Fosfomycin (J01XX)	*1st choice*: Nitrofurantoin (J01XE) ***5 days*** *2nd choice*: fosfomycin (J01XX) *3rd choice*: Trimethoprim (J01EA)
UTI with tissue invasion	Amoxicillin (J01CA)	*1st choice*: Amoxicillin-	*1st choice*: Amoxicillin-	*1st choice*: Ciprofloxacin

For peer review only

		clavulanic acid (J01CR)<br>*2nd choice*:<br>Sulfamethoxazole-trimethoprim (J01EE)	clavulanic acid (J01CR)<br>*2nd choice*:<br>Sulfamethoxazole-trimethoprim (J01EE) *or* Fluoroquinolone (J01MA)	(J01MA)<br>*2nd choice*:<br>Amoxicillin-clavulanic acid (J01CR)<br>*3rd choice*:<br>Sulfamethoxazole-trimethoprim (J01EE)
**Men**				
Cystitis	UTI in men should always be considered to be prostatitis	Nitrofurantoin (J01XE)<br>*or*<br>Trimethoprim (J01EA)	*1st choice*:<br>Nitrofurantoin (J01XE)<br>*2nd choice*:<br>Trimethoprim (J01EA)	*1st choice:*<br>Nitrofurantoin (J01XE)<br>*2nd choice*:<br>Trimethoprim (J01EA)
UTI with tissue invasion	Trimethoprim (J01EA) *or* Amoxicillin (J01CA)	*1st choice*:<br>Amoxicillin-clavulanic acid (J01CR)<br>*2nd choice*:<br>sulfamethoxazole-trimethoprim (J01EE)	*1st choice*:<br>Amoxicillin-clavulanic acid (J01CR)<br>*2nd choice*:<br>Sulfamethoxazole-trimethoprim (J01EE) *or* Fluorochinolone (J01MA)	*1st choice*:<br>Ciprofloxacin (J01MA)<br>*2nd choice*:<br>Amoxicillin-clavulanic acid (J01CR)<br>*3rd choice*:<br>Sulfamethoxazole-trimethoprim (J01EE)

The table shows the treatment of UTIs according to the Dutch guidelines of 1989, 1999, 2005 and 2013.[4-7] The recommended duration of treatment with nitrofurantoin is also described. Please note that information from guidelines on the treatment of pregnant women, and also the treatment of risk groups such as diabetes patients or abnormalities of the urinary tract differs is not described. Furthermore, the treatment duration of men is in all case longer than in women.

Previous research shows that the use of antimicrobial drugs in the Netherlands in general has increased since 2005 until approximately 2012, most prominently in elderly patients, but has decreased again since then.[8 9] Since UTIs are a major reason to consult a GP, use of antimicrobial drugs for UTIs contribute significantly to total antimicrobial consumption. Therefore, antibiotic stewardship focusing on (inappropriate) antimicrobial drug prescribing for UTIs is an important target in the fight against antimicrobial resistance (AMR). This study aims to study the choice of antimicrobial drugs prescribed for UTIs by GPs and the duration of nitrofurantoin use over time in relation to the Dutch national guidelines

For peer review only

(of 1989, 1999, 2005 and 2013) for the treatment of UTIs in primary care using a large electronic primary care database.

For peer review only

Materials and methods

Data source

This study was conducted using data from the Integrated Primary Care Information database (IPCI), which is a longitudinal observational dynamic database containing the records from more than 450 general practices throughout the Netherlands.[10] Briefly, IPCI contains the complete electronic medical records of ~2,500,000 patients, containing amongst others data on diagnoses (coded and free text) and prescriptions coded according to the ATC (anatomical therapeutical chemical) classification of the WHO.[11] The system complies with European Union guidelines on the use of data for medical research and has been proven valid for pharmaco-epidemiological studies. More detailed information on IPCI has been described elsewhere.[10] The scientific and ethical advisory board of IPCI approved this study (nr. 11/2015).

Study population

The study cohort comprised patients aged 12 years and older with at least one year of valid database history in the IPCI database. The study period was from the 1st of January 1996 until the 1st of January 2015. Follow-up started from the following, whichever occurred last: start of study period, age of 12 years, and reaching a minimum of 12 months of database history. Follow-up ended when a patient left the database, died or at the end of the study period, whichever occurred first. Gender, age at time of prescription(s) and follow-up time (time of each patient in the database) were assessed for all patients.

Prescriptions

From the database, we selected all prescriptions for antimicrobial drugs prescribed during the study period, using an automatic search on the ATC code "J01", which is the ATC code for antimicrobial drugs. All prescriptions of antimicrobial drugs were further categorized by ATC drug class e.g. J01AA

For peer review only

(tetracyclines). (**Table S1**) We analyzed all prescriptions of ATC class J01CA (penicillins with extended spectrum), J01CR (combinations of penicillins, incl. beta-lactamase inhibitors), J01EA (trimethoprim and derivatives), J01EE (combinations of sulfonamides and trimethoprim, incl. derivatives), J01MA (fluoroquinolones), J01XE (nitrofuran derivatives) and J01XX (other antimicrobials (mainly fosfomycin)). In addition, the duration of all prescriptions of nitrofurantoin (J01XE01) (which in the Netherlands is only prescribed for UTIs) was assessed.

Indication of use of antimicrobial drugs

Indication of use was assessed through an automatic search on disease specific codes. Antimicrobial drug prescriptions were linked to the indication of use through a unique patient identifier linking a prescription to a diagnosis using the International Classification of Primary Care (ICPC-1) codes (version 5). These ICPC codes were categorized in the following indications: UTIs, skin infections, airway infections, ear infections or other infections (**Table S2**). All prescriptions without an ICPC code were assigned to the group: 'no code for indication of use'. Urethritis was included in the group of "other" infections and not in the UTI group because of the distinct pathophysiology.

Analyses

The total number of prescriptions for UTIs, 'other infections' (including respiratory infections, skin infections, ear infections etc.) and prescriptions without an indication code were calculated for the complete study time and per year. Additionally, the proportion of UTI prescriptions per calendar year was calculated with as denominator all antimicrobial drug prescriptions with an indication code. Also, all nitrofurantoin prescriptions were analyzed per year.

Since both the number of users, but also the total number of antimicrobial drug prescriptions are interesting with regard to the study of antimicrobial resistance, we studied both. Because of the

For peer review only

dynamic nature of the study cohort, the annual frequency of antimicrobial drug prescriptions was calculated by dividing the total number of antimicrobial drug prescriptions by the total number of person-years (PY). The annual number of users per calendar year was calculated by dividing all users by the total number of PY in that specific year. With regard to the calculation of users, if an individual received prescriptions of more than one antimicrobial drug classes in one year, the individual contributed data to the different classes. However, if an individual received 2 or more prescriptions of the same drug in one year, the individual contributed only once as a user of this specific drug class. The frequency of prescriptions and users were studied by age (12-17, 18-25, 26-35, 36-45, 46-55, 56-65, 66-75, 76-85 and ≥86 years age categories), gender and calendar year. The prescribing of antimicrobial drugs by GPs was compared to the recommendations according to the national guidelines **(Table 1)**.[4-7]

We intended to investigate if improved coding over time has influenced the results of the frequency of prescribing antimicrobial drug prescriptions for UTIs in general, independent of the drug class. Therefore, we also studied the frequency of nitrofurantoin prescriptions over time (since nitrofurantoin was only prescribed for UTIs) and we studied the proportion of prescriptions for UTIs within total prescriptions with an indication code (thus including all antimicrobial drug prescriptions with information on indication of use).

Furthermore, all nitrofurantoin prescriptions prescribed to women during the study period were selected and duration of use was categorized in 3, 5, 7 days or in "other" (when the number of days of use was unknown or different to 3, 5, or 7 days). The proportion of each category with as denominator the total number of prescriptions of nitrofurantoin in women was calculated by year.

Finally, we used a time series ARIMA model (in SPSS 24) in order to determine the effect of the implementation of the guideline of 2005.[12] The calendar year was first univariably added to the analysis to study differences of antimicrobial drug prescribing over time. Next, we ran a model including calendar

For peer review only

year and the intervention (implementation of the guideline). When the p-value of the interaction term was < 0.05, the difference in slope before and after the implementation of the guideline was significant, implying a significant effect of the implementation of this guideline.

Patients and public involvement

No patients were involved in any stage of this study.

For peer review only

Results

The study population comprised 1,755,085 patients of 12 years and older with a mean follow-up time of 3.31 year. 671,251 (38.2%) patients were prescribed at least one antimicrobial drug during the study period; 271,772 (40.5%) men and 399,479 (59.5%) women. In total, they received 2,019,335 antimicrobial drug prescriptions (mean of 3.0 prescriptions per person) during the study period. Of these prescriptions, 1,144,810 (56.7%) could be linked to an indication, namely 528,464 (46.2%) antimicrobial drug prescriptions for airway infections; 401,655 (35.1%) for UTIs; 157,900 (13.8%) for skin infections; 29,984 (2.6%) for ear infections and 26,807 (2.3%) for "other" infections.

Antimicrobial drug prescriptions for UTIs by calendar year

For all years combined and only considering those prescriptions with indication of use, prescriptions for UTIs were 12.6% of the total prescriptions in men and 45.2% in women. The total number of prescriptions per year for UTIs increased from 9 (men) and 88 (women) prescriptions per 1,000 PY in 1996 to respectively 19 and 153 prescriptions per 1,000 PY in 2014. However, especially at the end of the study period, prescriptions without indication code decreased (**Figure 1)**. To control for improvement of coding over time, we studied the total number of nitrofurantoin prescriptions, which in the Netherlands is only prescribed for the indication UTI. In women more clearly than in men, we see a flattening or even decrease of the number of non-coded nitrofurantoin prescriptions, whereas the number of coded and total nitrofurantoin prescriptions increased **(Figure S1)**. Moreover, we calculated the proportion of antimicrobial drug prescriptions for UTI with as denominator all antimicrobial drug prescriptions (for all indications) with an indication code. In this analysis, the proportion of antimicrobial drugs for UTIs increased from 5.2% in 1996 to 14% in 2014 for men and from 28% in 1996 to 50% in 2014 for women **(Figure S2)**.

For peer review only

Fluoroquinolones (J01MA) are the most commonly prescribed antimicrobial drugs in men with UTIs with no clear increase or decrease over time. Other frequently prescribed antimicrobial drugs were combinations of sulfonamides and trimethoprim (J01EE) and combinations of penicillins, incl. beta-lactamase inhibitors (J01CR), the last group increased significantly over time until 2013. Also, in men, the number of nitrofuran derivatives prescriptions significantly increased from 0.4 prescriptions per 1,000 PY in 1996 to 6.2 prescriptions per 1,000 PY in 2014. In women, nitrofuran derivatives (J01XE) were clearly the most frequently prescribed drugs since 1999, with a strong and significant increase in the last years from 52 prescriptions per 1,000 PY in 2008 to 98 prescriptions per 1,000 PY in 2014. They increasingly replaced the prescriptions of trimethoprim and derivatives (J01EA) and fluoroquinolones (J01MA), which were also commonly prescribed in women. Also, the prescriptions of combinations of penicillins, incl. beta-lactamase inhibitors (J01CR) and fosfomycin significantly increased, mainly in most recent years (**Figure 2**).

Choice of antimicrobial drug prescription in relation to the recommendations of the national primary care guidelines

The implementation of the Dutch guideline on the treatment of UTI of 2005 was associated in women with a significant decrease of the slope of prescriptions of trimethoprim and derivatives (J01EA) and an increase in nitrofuran derivatives (J01XE) prescriptions. Unfortunately, we did not have sufficient data before 1999 and after 2013 to study the implementation of the other guidelines. For men, no significant effects were found of the implementation of the guidelines on the slope of any prescriptions.

Prescriptions of antimicrobial drugs for UTIs by age groups

Both in men and women, an increase in total number of UTI prescriptions was observed by age category. In women the number of prescriptions in all age groups is higher than in men, with nitrofuran derivatives (J01XE) as the most prescribed antimicrobial drug in all age groups, followed by

For peer review only

fluoroquinolones (J01MA) and trimethoprim and derivatives (J01EA). In men, fluoroquinolones (J01MA) were the most frequently prescribed antimicrobial drugs in most age groups, followed by nitrofuran derivatives (J01XE) and combinations of penicillins, incl. beta-lactamase inhibitors (J01CR) **(Figure 3)**.

Analyzing the total number of prescriptions for UTIs (J01 – antibacterials for systemic use) per age category, we observed an increase in the last years for all age categories, but most prominent in the higher age categories **(Figure S3)**.

Changes in duration of nitrofurantoin prescriptions

In total, 215,531 prescriptions of nitrofurantoin with an ICPC code for UTIs were prescribed to women in the study period. In line with a change in recommended duration from 3 to 5 days in 2005, the proportion of prescriptions with a duration of 5 days increased strongly from 2005 onwards, while proportion of prescriptions with a duration of 3 days became scarce. The proportion of prescriptions with a duration of 7 days decreased from 33% in 1996 to 18% in 2014. The proportion of prescriptions with a duration other than 3, 5 or 7 days (defined as “other duration”) remained stable over time, with a proportion ranging between 6% and 10% **(Figure 4)**.

For peer review only

Discussion

In this study, we investigated the prescribing of antimicrobial drugs for UTIs in primary care during the study period January 1st 1996 until January 1st 2015 and compare this with the recommendations in the Dutch primary care guidelines. These guidelines are actively distributed by the Dutch College of General Practitioners.

During the study period, nitrofurantoin was shown to be the most prescribed drugs for the treatment of UTIs, especially in women, and increasingly in men. The Dutch guidelines of 1989 indicated nitrofurantoin as one of the options for women, whereas it was suggested as option for both men and women in the guideline of 1999 and as first-choice drug for cystitis in the 2005 and 2013 guideline. This is reflected in our findings; we observed in men and women an increase in nitrofurantoin prescriptions since 1999, with a steep and significant increase since 2005. In this period nitrofurantoin replaced trimethoprim in women, which significantly decreased after 2005. Furthermore, in the guidelines of 1999 and 2005, amoxicillin-clavulanic acid was recommended for UTIs with tissue invasion, whereas amoxicillin (or trimethoprim in men) was recommended for UTIs with tissue invasion in the guidelines prior to 1999. In men and women, a significant increase of combinations of penicillins, incl. beta-lactamase inhibitors such as amoxicillin-clavulanic acid, was shown during the study period. In men, a flattening was seen from 2012 on, just before the guideline of 2013, which recommended ciprofloxacin as first-choice.

We also studied the compliance of GPs to guidelines with regard to the duration of nitrofurantoin prescribing in women. The recommended duration increased from 3 to 5 days in 2005, which is reflected in our findings, indicating an increase of adherence to the guidelines by GPs, mainly since the guidelines of 2005 and 2013.[4-7] A deviation from the UTI guidelines was observed with regard to the prescribing of fluoroquinolones. Fluoroquinolones, in particular ciprofloxacin, were only advised

For peer review only

as first-choice drug for UTIs with tissue invasion from 2013 on (in 2005 it was 2nd choice and in 1989 use was discouraged), whereas in men these drugs were the most frequently described antimicrobial drug during the complete study period. In women, we saw a significant decrease over time suggesting an increase of compliance of GPs over time.

A study in another Dutch GP database investigated antimicrobial prescribing for several infections during 2001 and showed that approximately 75% of the prescriptions for cystitis were first-choice drugs (nitrofurantoin or trimethoprim) according to guidelines.[13] The percentage of first-choice drug prescriptions in our study in 2001 is comparable (approximately 67% for women). A smaller study from 2009 in 970 non-pregnant women of 11 years and older with cystitis determined that 66% were prescribed nitrofurantoin, whereas we found a proportion of 57% in all women in 2009.[14] Our proportion is somewhat smaller, possibly because we did not distinguish between cystitis and pyelonephritis, since a proportion of the prescriptions had an indication code for both. However, the sample size of the other study is much smaller resulting in more uncertainty around the true prevalence of antimicrobial drug use.

As part of new health quality standards, GPs are encouraged to improve disease coding, a phenomenon also observed in other countries. This might in part explain the increase of total prescriptions for UTIs which is underlined by our finding that the number of prescriptions for nitrofurantoin without an indication code seemed to decrease, especially in women since approximately 2007. To control for this bias, we analyzed UTI prescriptions as a proportion of the total number of antimicrobial drug prescriptions with an indication code (thus excluding prescriptions without a disease code). Yet, we observed that the increase of prescriptions was larger for UTI's than for other indications. This increase in antimicrobial drug prescribing for the treatment of UTI is supported by other literature. According to the NIVEL (Netherlands institute for health services research) registration, the number of contacts with a

For peer review only

GP for UTIs increased (especially in women) from 185 per 1,000 in 2012 to 295 per 1,000 in 2015.[3] This was confirmed by another large Dutch GP database, showing an increase of GP visits for UTIs and antimicrobial drug prescriptions for UTI in the period 2007-2010.[15 16] This increase of use of antimicrobial drugs for UTIs might partly be explained by ageing of the population. Indeed, we showed that antimicrobial drugs were especially prescribed to patients in older age categories. Additionally, a Dutch study from 2012 also showed an increase of antimicrobial drug prescribing by age, and an increase of total prescriptions of antimicrobial drugs to elderly: in 2000, 9% of patients above 80 years had at least one prescription, which increased to 22% in 2009.[8] However, ageing of the population cannot solely explain the increase, since we also showed an increase in prescriptions of antimicrobial drugs in younger age groups. Possible explanations might be that more patients suffer from recurrent UTIs, that patients more frequently visit their GP in case of a UTI or that GPs more easily prescribe antimicrobial drugs for UTIs, but this has to be elucidated in future studies.

One of the strengths of this study is that we use a large population-based cohort from a database with detailed information on prescriptions and where information is collected as part of routine clinical care reducing the risk of selection and information bias. We used these data to show that they can be of importance in studying antibiotic stewardship. Unfortunately, because of confidentiality, we could not study differences in prescribing patterns by region nor by GP characteristics such as age and/or gender. Additionally, although we found that antimicrobial drugs were increasingly prescribed according to the guidelines, the study design did not allow us to investigate the reasons behind deviations from the guidelines. This is of interest and should be studied further, eventually by qualitative prospective research to distinguish between forced deviations caused by resistant pathogens and other deviations, which should be diminished to promote good antimicrobial stewardship.

For peer review only

Reducing the use of antimicrobial drugs is important, since the drawback of treating infections with antimicrobial drugs is the development of AMR.[17] Patients suffering from infections caused by resistant bacteria have an increased risk of a worse clinical outcome and death. [17] Moreover, AMR leads to an increase in healthcare costs, which has been estimated to be 1.5 billion euros in EU every year.[18 19] Misuse and overuse of antimicrobial drugs are the most important risk factors of antimicrobial resistance worldwide.[20] Therefore, antimicrobial stewardship programs have been developed, aiming to optimize the prescribing of antimicrobial drugs, minimize misuse and overuse in order to minimize AMR.[21] Our study demonstrates that databases with antimicrobial drug prescriptions and indications are useful for surveillance of antimicrobial drug prescriptions over time. In future, information gathered in such databases should be provided to GP practices as part of antibiotic stewardship in primary care.

Funding

The authors have no funding to report.

Competing interests statement

KV works for a department who in the past received unconditional grants from: Pfizer/BI, Yamanouchi, GSK, Novartis. None of which are related to the content of this paper. The other authors have nothing to declare.

Author’s contributions

MM, AV, BS and KV designed the study. MM, EB and KV analyzed the data and interpreted these with the aid of AV and BS. MM wrote the manuscript with critical input from all co-authors.

Data sharing statement

For peer review only

The aggregated data are available with the corresponding author upon reasonable request.

For peer review only

References

1. Foxman B. Epidemiology of urinary tract infections: incidence, morbidity, and economic costs. Am J Med 2002;**113 Suppl 1A**:5S-13S.
2. Foxman B. The epidemiology of urinary tract infection. Nat Rev Urol 2010;**7**(12):653-60.
3. NIVEL Zorgregistraties eerste lijn 2015: Nederlands instituut voor onderzoek van de gezondheidszorg; [updated 02-02-2016. Available from: https://www.nivel.nl/nl/NZR/huisarts-top-20-diagnoses-bij-contacten-naar-geslacht].
4. van Pinxteren BK, B. J.; Geerlings, S. E.; Visser, H. S., Klinkhamer, S.; van der Weele, G. M.; Verduijn, M. M.; Opstelten, W.; Burgers, J. S.; van Asselt, K. M. NHG-Standaard Urineweginfecties. Huisarts en Wetenschap 2013;**56**(6):270-80.
5. Timmermans AE, Baselier PJAM, Winkens RAG, et al. NHG-Standaard Urineweginfecties. Huisarts en Wetenschap 1999;**42**(13):613-22.
6. van Balen FAM, Baselier PJAM, van Pienbroek E, et al. NHG-Standaard Urineweginfecties Huisarts en Wetenschap 1989;**32**(11):439-43.
7. van Haaren KAMV, H. S.; van Vliet, S.; Timmermans, A. E.; Yadava, R.; Geerlings, S. E.; ter Riet, G.; van Pinxteren, B. NHG-Standaard Urineweginfecties Huisarts en Wetenschap 2005;**48**(7):341-52.
8. Haeseker MB, Dukers-Muijrers NH, Hoebe CJ, et al. Trends in antibiotic prescribing in adults in Dutch general practice. PLoS One 2012;**7**(12):e51860.
9. NethMap. Consumption of antimicrobial agents and antimicrobial resistance among medically important bacteria in The Netherlands in 2015: SWAB/RIVM, 2016.
10. Vlug AE, van der Lei J, Mosseveld BM, et al. Postmarketing surveillance based on electronic patient records: the IPCI project. Methods Inf Med 1999;**38**(4-5):339-44.
11. WHO Collaborating Centre for Drug Statistics Methodology. [Available from: https://www.whocc.no/].
12. Cochrane Effective Practice and Organisation of Care. Interrupted time series (ITS) analyses: EPOC Resources for review authors; 2017 [Available from: http://epoc.cochrane.org/epoc-specific-resources-review-authors].
13. Ong DS, Kuyvenhoven MM, van Dijk L, et al. Antibiotics for respiratory, ear and urinary tract disorders and consistency among GPs. J Antimicrob Chemother 2008;**62**(3):587-92.
14. den Heijer DD, GA. Maes, J. Stobberingh, EE. Antibiotica bij ongecompliceerde urineweginfecties: geen toename van resistentie in de afgelopen 5 jaar. Nederlands Tijdschrift voor Geneeskunde 2011;**155**.
15. Willems CS, van den Broek D'Obrenan J, Numans ME, et al. Cystitis: antibiotic prescribing, consultation, attitudes and opinions. Fam Pract 2014;**31**(2):149-55.
16. van den Broek d'Obrenan J, Verheij TJ, Numans ME, et al. Antibiotic use in Dutch primary care: relation between diagnosis, consultation and treatment. J Antimicrob Chemother 2014;**69**(6):1701-7.
17. Bergman M, Nyberg ST, Huovinen P, et al. Association between antimicrobial consumption and resistance in Escherichia coli. Antimicrob Agents Chemother 2009;**53**(3):912-7.
18. Cosgrove SE. The relationship between antimicrobial resistance and patient outcomes: mortality, length of hospital stay, and health care costs. Clin Infect Dis 2006;**42 Suppl 2**:S82-9.
19. The bacterial challenge: time to react. A call to narrow the gap between multidrug-resistant bacteria in the EU and the development of new antibacterial agents: ECDC/EMEA Joint technical report, 2009.
20. Ventola CL. The antibiotic resistance crisis: part 1: causes and threats. P T 2015;**40**(4):277-83.

For peer review only

21. Ventola CL. The antibiotic resistance crisis: part 2: management strategies and new agents. P T 2015;**40**(5):344-52.

Figure 1: The number of users and prescriptions of antimicrobial drugs for UTIs, other infections and infections without indication code in the period 1996 – 2014.

Figure 2: The number of users and prescriptions of antimicrobial drugs for urinary tract infections in the period 1996 – 2014. Note that the scale of the y-axis differs between men and women.

Figure 3: The number of users and prescriptions of antimicrobial drugs for urinary tract infections per age group. Note that the scale of the y-axis differs between men and women.

Figure 4: The proportion of the different durations of nitrofurantoin prescriptions in women in the period 1996 – 2014. The proportion of prescriptions of nitrofurantoin prescriptions (J01XXE01) with a duration of 3, 5, 7 days or "other" duration (unequal to 3, 5, 7 days or unknown) with in de denominator all these prescriptions. Until 2005 the duration recommended by the guideline was 3 days, whereas since guideline of 2005 the advised duration was 5 days.

For peer review only

For peer review only

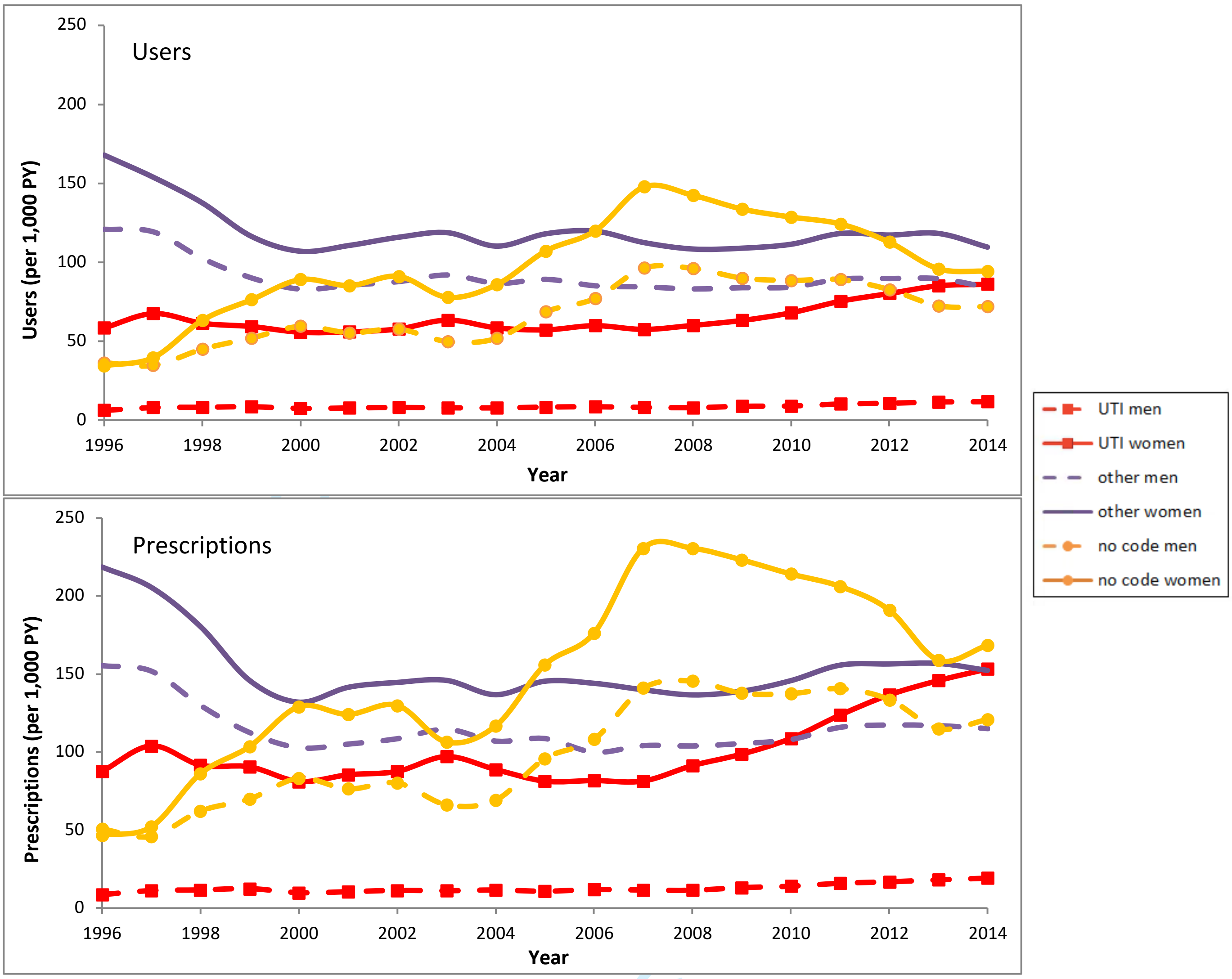

Figure 1: The number of users and prescriptions of antimicrobial drugs for UTIs, other infections and infections without indication code in the period 1996 – 2014.

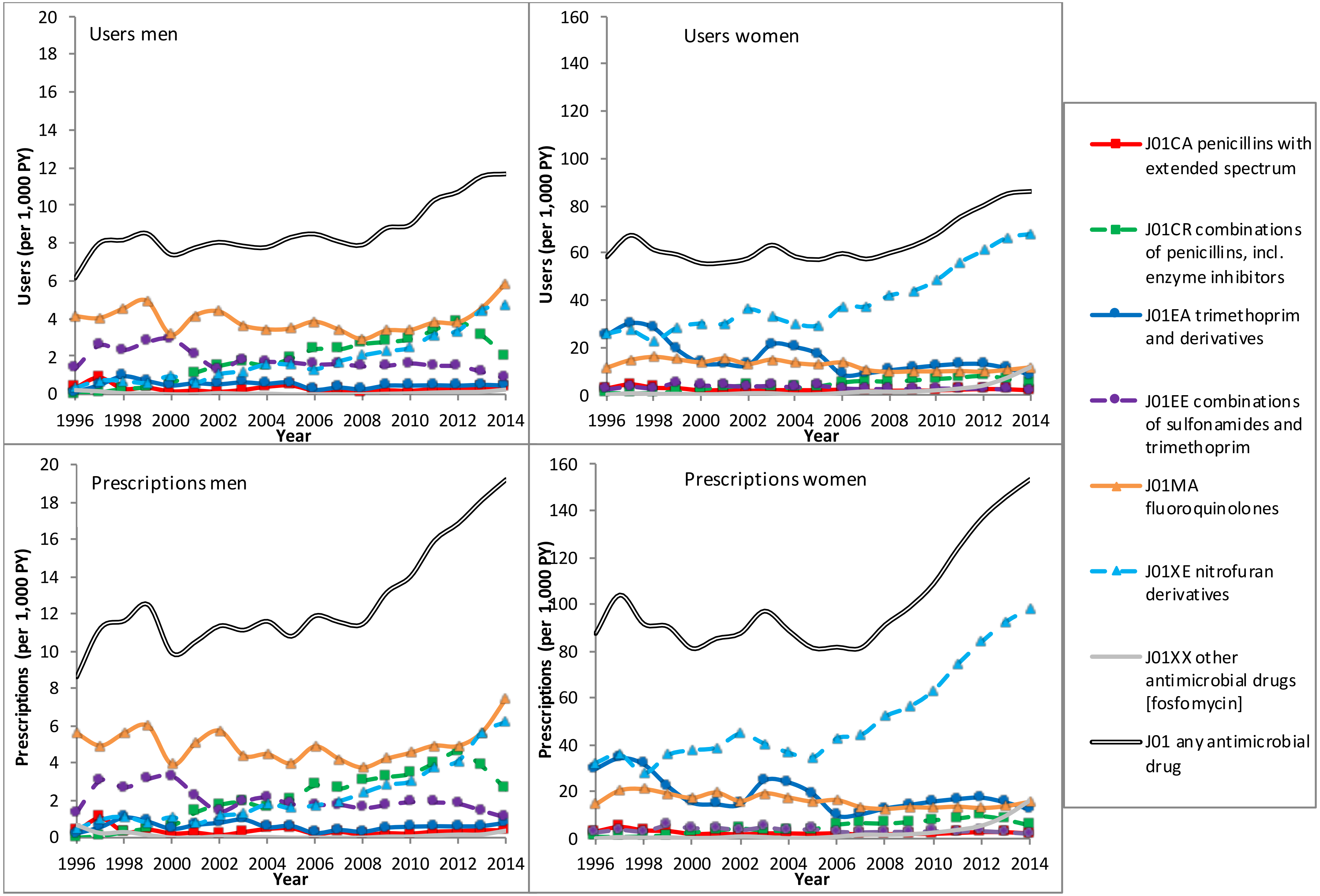
Users men
Users women
Prescriptions men
Prescriptions women
Users (per 1,000 PY)
Prescriptions (per 1,000 PY)
Year
J01CA penicillins with extended spectrum
J01CR combinations of penicillins, incl. enzyme inhibitors
J01EA trimethoprim and derivatives
J01EE combinations of sulfonamides and trimethoprim
J01MA fluoroquinolones
J01XE nitrofuran derivatives
J01XX other antimicrobial drugs [fosfomycin]
J01 any antimicrobial drug

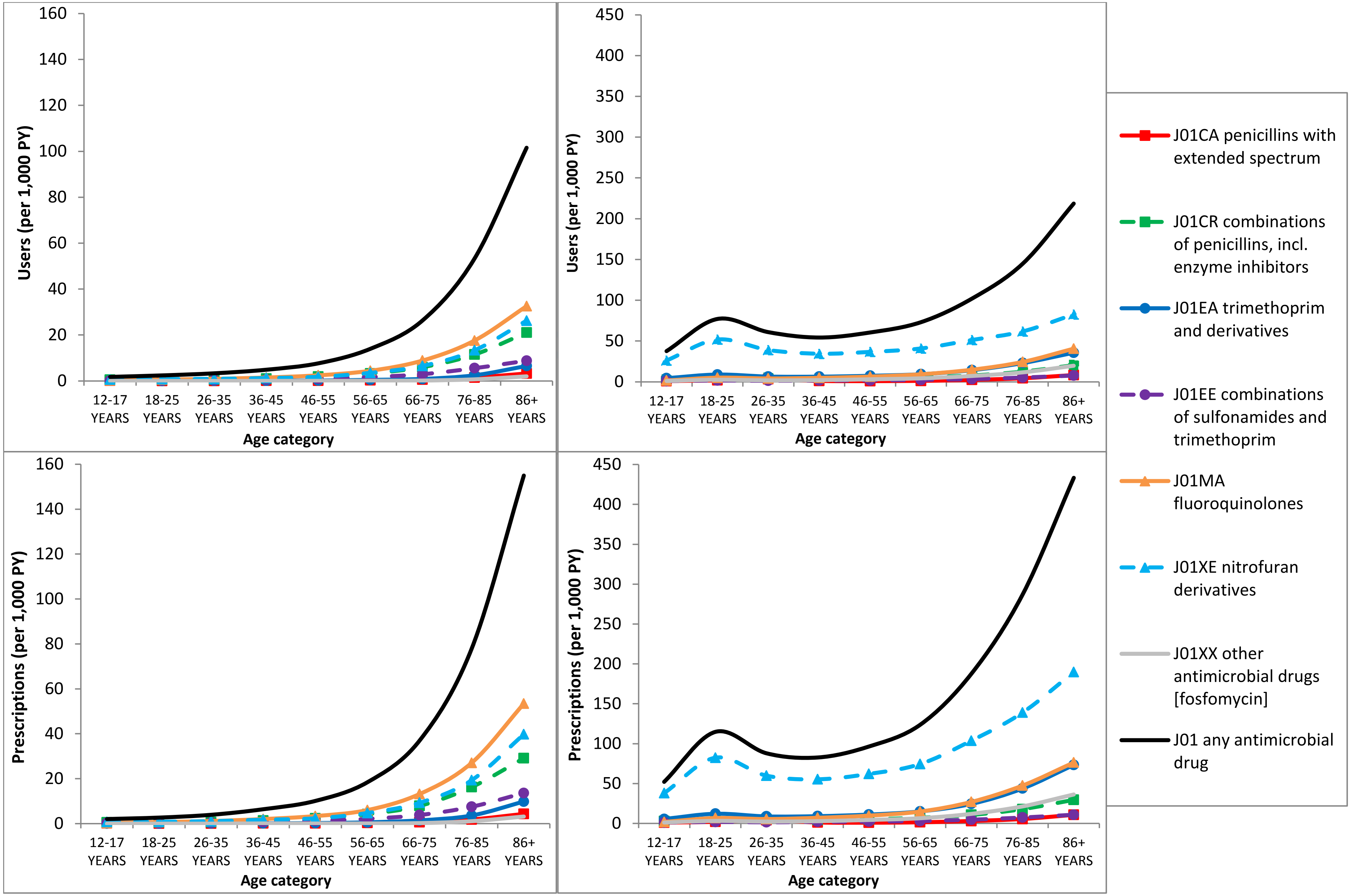
Users (per 1,000 PY)
Prescriptions (per 1,000 PY)
Age category
12-17 YEARS
18-25 YEARS
26-35 YEARS
36-45 YEARS
46-55 YEARS
56-65 YEARS
66-75 YEARS
76-85 YEARS
86+ YEARS
0
20
40
60
80
100
120
140
160
50
150
200
250
300
350
400
450
J01CA penicillins with extended spectrum
J01CR combinations of penicillins, incl. enzyme inhibitors
J01EA trimethoprim and derivatives
J01EE combinations of sulfonamides and trimethoprim
J01MA fluoroquinolones
J01XE nitrofuran derivatives
J01XX other antimicrobial drugs [fosfomycin]
J01 any antimicrobial drug

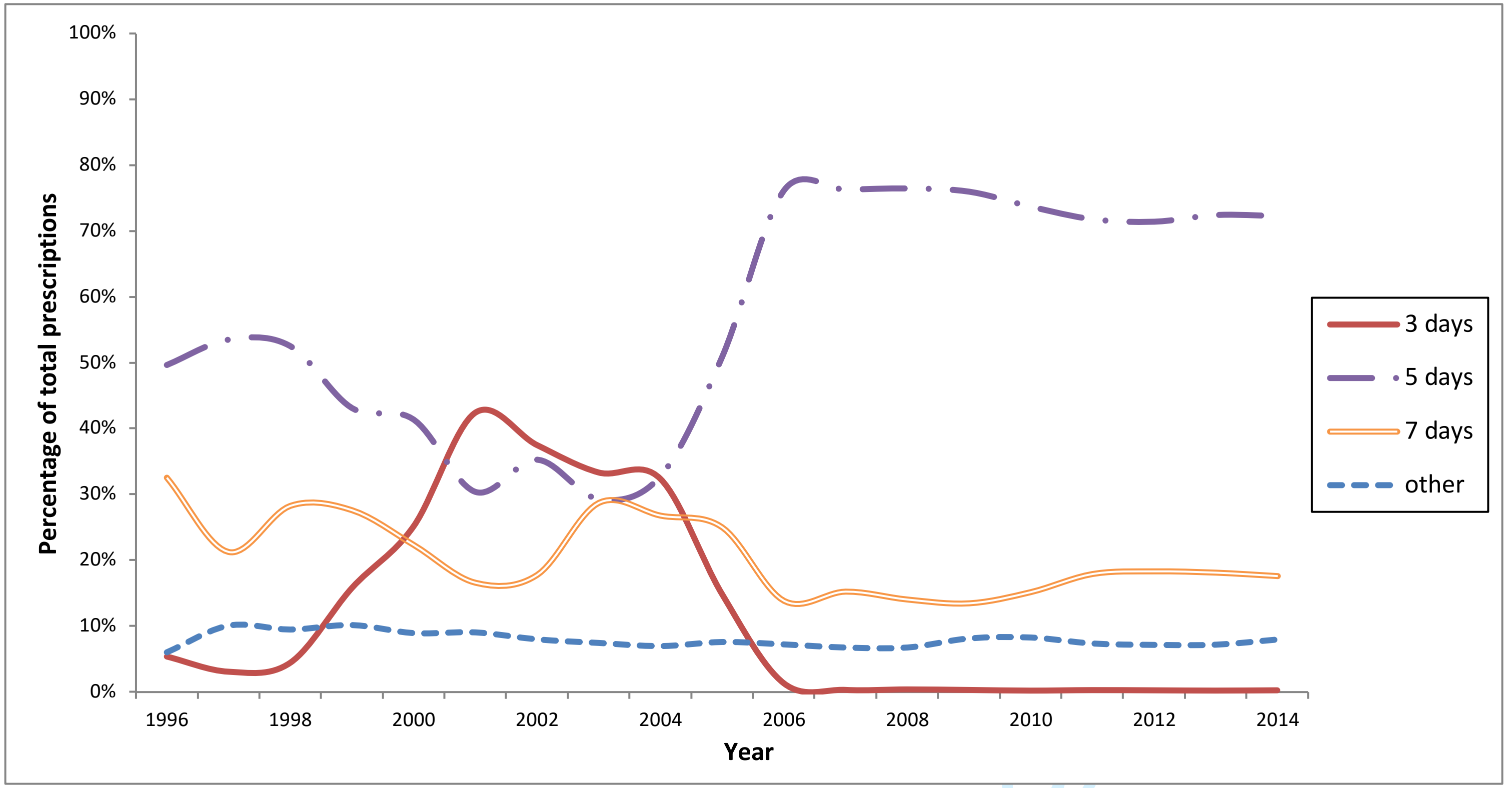

Figure 4: The proportion of the different durations of nitrofurantoin prescriptions in women in the period 1996 – 2014. The proportion of prescriptions of nitrofurantoin prescriptions (J01XXE01) with a duration of 3, 5, 7 days or "other" duration (unequal to 3, 5, 7 days or unknown) with in de denominator all these prescriptions. Until 2005 the duration recommended by the guideline was 3 days, whereas since guideline of 2005 the advised duration was 5 days.

Supplementary table 1: ATC codes of antimicrobial drugs prescribed by GPs to treat urinary tract infections

ATC code	Drug class
J01AA*	Tetracyclines
J01CA	Penicillins with extended spectrum
J01CE*	Beta-lactamase sensitive penicillins
J01CF*	Beta-lactamase resistant penicillins
J01CR	Combinations of penicillins, incl. beta-lactamase inhibitors
J01DB*	First-generations cephalosporins
J01DC*	Second-generation cephalosporins
J01DD*	Third-generation cephalosporins
J01EA	Trimethoprim and derivatives
J01EB*	Short-acting sulfonamides
J01EE	Combinations of sulfonamides, and trimethoprim, incl. derivatives
J01FA*	Macrolides
J01FF*	Lincosamides
J01GB*	Other aminoglycosides
J01MA	Fluoroquinolones
J01MB*	Other quinolones
J01XC*	Steroid antibacterials
J01XE	Nitrofuran derivatives
J01XX	Other antibacterials

All antimicrobial drug classes that were prescribed for UTIs in the study population. The number of prescriptions of drugs with * was very low and are not shown in the figures.

For peer review only

Supplementary table 2: ICPC codes

ICPC code	Indication
A03	Fever
A78	Lyme disease
A99	Other generalized/not specified diseases
B70	Acute lympadenitis
D70	Infectious diarrhea
D73	Supposititious gastroenteritis
F70	Infectious conjunctivitis
F72	Blepharitis
F73	Other infection/infection eye/adnexa
H04	Excretion from ear
H70	Otitis externa
H71	Acute otitis media
H72	Otits media with effusion
H74	Chronic otitis media/mastoiditis/other infection
R02	Dyspnoea attributed to airways
R05	Cough
R09	Symptoms/complaints sinuses
R21	Sore throat
R22	Symptoms/complaints tonsils
R70	Tuberculosis (airways)
R71	Pertussis
R72	Scarlet fever
R74	Cold/pharyngitis
R75	Acute/chronic rhinosinusitis
R76	Acute tonsillitis/peritonsillar abscess
R77	Acute laryngitis/tracheitis
R78	Acute bronchitis
R81	Pneumoniae
R83	Other respiratory infections
R90	Hypertrophy/chronic infection tonsils
S06	Local redness/erythema skin
S09	Local infection finger/toe/paronychia
S10	Furuncle/karbunkel/local cellulitis
S11	Folliculitis

For peer review only

S12	Tick bite
S13	Bite human/animal
S18	Cut
S76	Other skin infection
S84	Impetigo
S87	Constitutional eczema
S88	Other eczema
S96	Acne
U01	Painful micturition*
U02	Frequent micturition*
U70	Acute pyelonephritis*
U71	Cystitis*
U72	Urethritis
U88	Glomerulonephritis
X84	Vaginitis
X99	Other disease of female reproduction organs
W94	Mastitis
Y74	Orchitis/Epididymitis
Y75	Balanitis

All indications that were used in the study as possible indications for which antimicrobial drugs were prescribed. Antimicrobial drugs prescribed for the indications with * were assigned as antimicrobial drugs prescribed for UTIs.

For peer review only

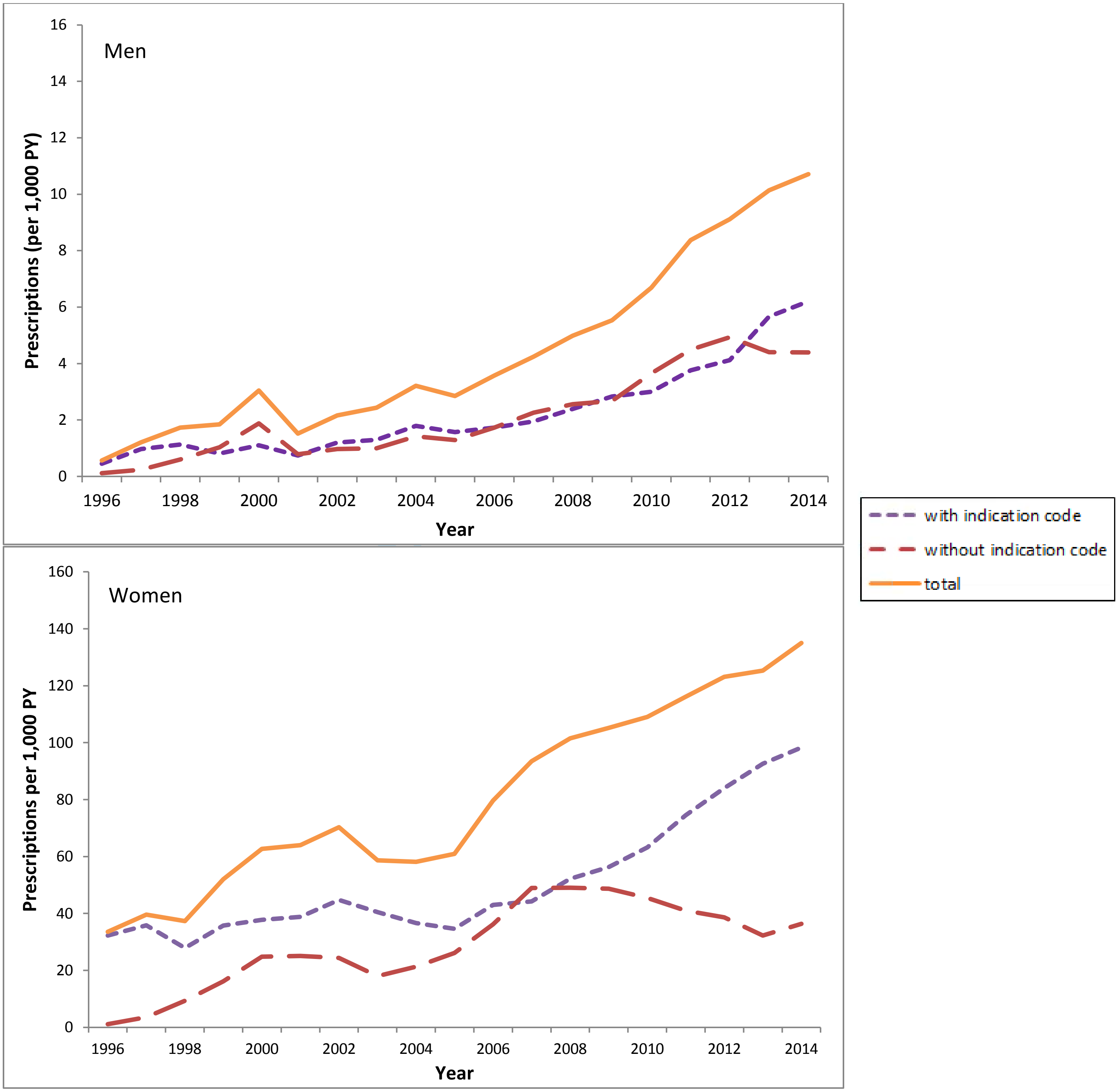

Supplementary figure 1: The number of prescriptions of nitrofurantoin with and without an indication code for urinary tract infections for men and women. Note that the scale of the y-axis differs between men and women.

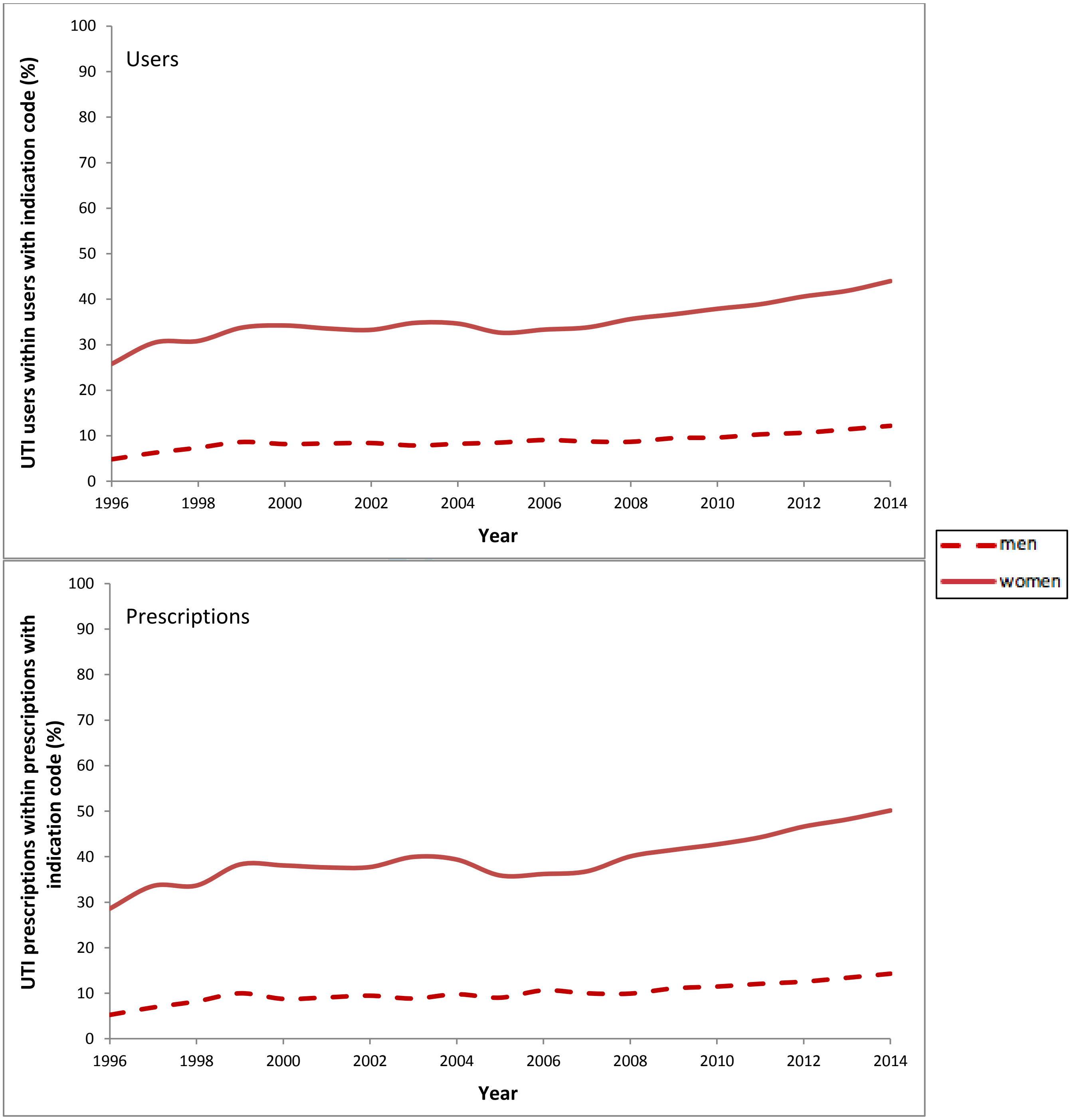

Supplementary figure 2: The percentage of users/prescriptions with an indication code for UTIs within all users/prescriptions with an indication code (thus including indication codes for UTIs, airway infections, ear infections, skin infections etc.).

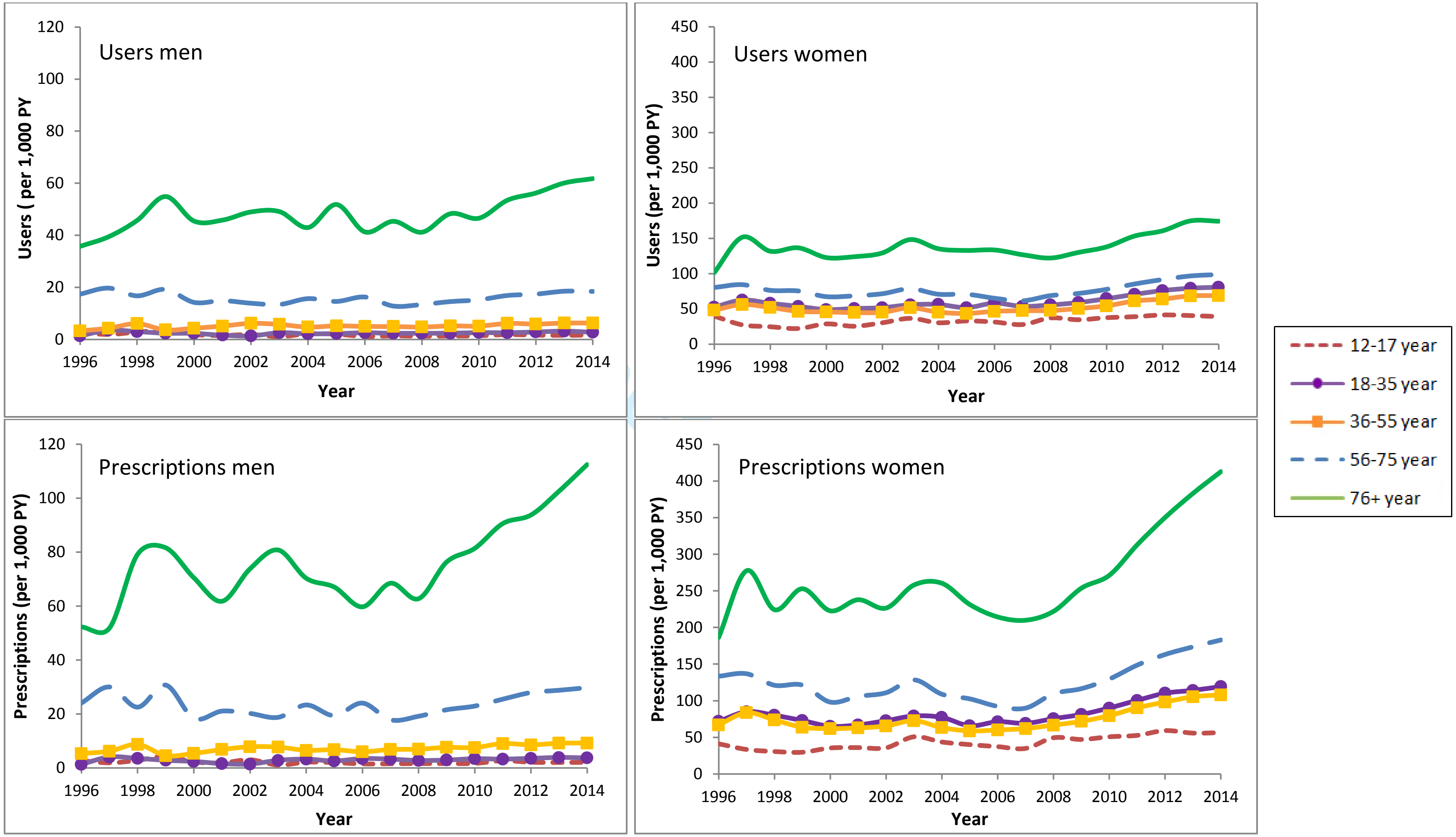

Supplementary figure 3: The number of users and prescriptions of antimicrobial drugs for urinary tract infections for different age groups per year. Note that the scale of the y-axis differs between men and women.

For peer review only

STROBE Statement—checklist of items that should be included in reports of ~~ob~~observational studies

Trends of prescribing antimicrobials drugs for UTIs in primary care in the Netherlands

	Item No	Recommendation	
**Title and abstract**	1	(*a*) Indicate the study's design with a commonly used term in the title or the abstract	✓ p1
		(*b*) Provide in the abstract an informative and balanced summary of what was done and what was found	✓ p2
**Introduction**			
Background/rationale	2	Explain the scientific background and rationale for the investigation being reported	✓ p4
Objectives	3	State specific objectives, including any prespecified hypotheses	✓ p4
**Methods**			
Study design	4	Present key elements of study design early in the paper	✓ p6
Setting	5	Describe the setting, locations, and relevant dates, including periods of recruitment, exposure, follow-up, and data collection	✓ p6/p7
Participants	6	(*a*) *Cohort study*—Give the eligibility criteria, and the sources and methods of selection of participants. Describe methods of follow-up<br>*Case-control study*—Give the eligibility criteria, and the sources and methods of case ascertainment and control selection. Give the rationale for the choice of cases and controls<br>*Cross-sectional study*—Give the eligibility criteria, and the sources and methods of selection of participants	✓ p6/p7
		(*b*) *Cohort study*—For matched studies, give matching criteria and number of exposed and unexposed<br>*Case-control study*—For matched studies, give matching criteria and the number of controls per case	NA
Variables	7	Clearly define all outcomes, exposures, predictors, potential confounders, and effect modifiers. Give diagnostic criteria, if applicable	✓ p6-8
Data sources/ measurement	8*	For each variable of interest, give sources of data and details of methods of assessment (measurement). Describe comparability of assessment methods if there is more than one group	✓ p6-8
Bias	9	Describe any efforts to address potential sources of bias	✓ p6-8 p9-10
Study size	10	Explain how the study size was arrived at	✓ p6/7
Quantitative variables	11	Explain how quantitative variables were handled in the analyses. If applicable, describe which groupings were chosen and why	✓ p6-8
Statistical methods	12	(*a*) Describe all statistical methods, including those used to control for confounding	✓ p8
		(*b*) Describe any methods used to examine subgroups and interactions	
		(*c*) Explain how missing data were addressed	
		(*d*) *Cohort study*—If applicable, explain how loss to follow-up was addressed<br>*Case-control study*—If applicable, explain how matching of cases and controls was addressed<br>*Cross-sectional study*—If applicable, describe analytical methods taking account of sampling strategy	
		(*e*) Describe any sensitivity analyses	

Continued on next page

For peer review only - http://bmjopen.bmj.com/site/about/guidelines.xhtml

**Results**			
Participants	13*	(a) Report numbers of individuals at each stage of study—eg numbers potentially eligible, examined for eligibility, confirmed eligible, included in the study, completing follow-up, and analysed	✓ P9
		(b) Give reasons for non-participation at each stage	
		(c) Consider use of a flow diagram	
Descriptive data	14*	(a) Give characteristics of study participants (eg demographic, clinical, social) and information on exposures and potential confounders	✓ P9
		(b) Indicate number of participants with missing data for each variable of interest	
		(c) *Cohort study*—Summarise follow-up time (eg, average and total amount)	✓ P9
Outcome data	15*	*Cohort study*—Report numbers of outcome events or summary measures over time	✓ P9
		Case-control study—Report numbers in each exposure category, or summary measures of exposure	
		Cross-sectional study—Report numbers of outcome events or summary measures	
Main results	16	(*a*) Give unadjusted estimates and, if applicable, confounder-adjusted estimates and their precision (eg, 95% confidence interval). Make clear which confounders were adjusted for and why they were included	NA
		(*b*) Report category boundaries when continuous variables were categorized	P9-11
		(*c*) If relevant, consider translating estimates of relative risk into absolute risk for a meaningful time period	
Other analyses	17	Report other analyses done—eg analyses of subgroups and interactions, and sensitivity analyses	✓ P9-11
**Discussion**			
Key results	18	Summarise key results with reference to study objectives	✓ P12
Limitations	19	Discuss limitations of the study, taking into account sources of potential bias or imprecision. Discuss both direction and magnitude of any potential bias	✓ P13/14
Interpretation	20	Give a cautious overall interpretation of results considering objectives, limitations, multiplicity of analyses, results from similar studies, and other relevant evidence	✓ P12-14
Generalisability	21	Discuss the generalisability (external validity) of the study results	✓ P15
**Other information**			
Funding	22	Give the source of funding and the role of the funders for the present study and, if applicable, for the original study on which the present article is based	✓ P15

*Give information separately for cases and controls in case-control studies and, if applicable, for exposed and unexposed groups in cohort and cross-sectional studies.

Note: An Explanation and Elaboration article discusses each checklist item and gives methodological background and published examples of transparent reporting. The STROBE checklist is best used in conjunction with this article (freely available on the Web sites of PLoS Medicine at http://www.plosmedicine.org/, Annals of Internal Medicine at http://www.annals.org/, and Epidemiology at http://www.epidem.com/). Information on the STROBE Initiative is available at www.strobe-statement.org.
